# Robust ghost marker rejection in marker-based optical tracking using contour-derived monocular depth priors

**DOI:** 10.1371/journal.pone.0361058

**Published:** 2026-11-13

**Authors:** Enzan Chen, Rougang Zhou, Ange Wang, Chou Jay Tsai Chien, Junjie Zhu, Zhenchao Ruan

**Affiliations:** 1 School of Mechanical Engineering, Hangzhou Dianzi University, Hangzhou, China; 2 Wenzhou Institute of Hangzhou Dianzi University, Wenzhou, China; 3 Mstar Technologies, Inc., Hangzhou, China; 4 Department of Geriatrics, the First Affiliated Hospital, School of Medicine, Zhejiang University, Hangzhou, China; 5 Chongqing Railway Transport Technician College, Shapingba, Chongqing, China; 6 Shanghai Starriver Bilingual School, Shanghai, China; Polytechnic Institute of Leiria: Instituto Politecnico de Leiria, PORTUGAL

## Abstract

Marker-based stereo optical tracking can be disrupted by reflective interference and ambiguous correspondences when marker projections lie near the same epipolar line. We developed a tracking method that combines contour-based marker detection and constrained ellipse fitting with contour-derived monocular depth priors, uncertainty-normalized stereo matching, and adaptive region-of-interest (ROI) processing. In the reprojection-consistency evaluation, the proposed localization method achieved mean errors of 0.0532 and 0.0552 pixels in the left and right cameras, respectively, compared with 0.0827 and 0.0843 pixels for grayscale-centroid localization. In a separate inter-marker distance experiment involving 150 stereo observations, its mean absolute error was 0.0458 mm, compared with 0.0515 mm for OpenCV fitEllipse and 0.0551 mm for the grayscale-centroid method. In 50 stereo frames with deliberately induced ghost-marker ambiguity, the proposed matching method met the predefined frame-level correctness criterion in all 50 frames, compared with 20 frames for epipolar-only matching and 37 frames for MSRC. All expected markers were detected without false positives in the 100 images acquired for the paired illumination experiment. With 1–15 markers, adaptive ROI processing maintained a mean algorithmic processing time below 20 ms per stereo frame pair; at 15 markers, the mean was 16.52 ms and the 95th percentile was 21.20 ms. These results demonstrate improved measurement and matching performance under the tested laboratory conditions.

## Introduction

Traditional surgical procedures rely heavily on surgeons’ clinical experience and visual judgment. These procedures are not only time-consuming and technically demanding, but also struggle to meet the stringent requirements of modern minimally invasive surgery (MIS) for minimized incisions and rapid postoperative recovery. Against this backdrop, Optical Tracking Systems (OTS) have emerged and been widely applied in high-precision image-guided surgeries, including Parkinson’s disease treatment, neurosurgery, orthopedic surgery, and radiation therapy [1–6]. A typical OTS generally consists of dual cameras equipped with near-infrared (NIR) filters and a ring of infrared light sources [7]. By capturing reflective markers attached to medical instruments or the patient’s surface, the system achieves high-precision three-dimensional spatial localization of targets based on the principle of triangulation. During surgical procedures, this system can track instrument movements and patient posture variations in real time [8], providing surgeons with precise intraoperative navigation.

However, the core prerequisite for high-precision localization is that the binocular cameras must perform unambiguous recognition and correspondence matching of marker projections on the image planes. Reflective markers used in clinical and laboratory tracking systems are typically small, visually similar, and weakly textured; therefore, stereo matching based only on grayscale correlation or local image features is often unreliable. In scenarios involving multiple instruments or densely distributed markers, overlap or alignment of marker projections can produce binocular matching ambiguities and false “ghost markers” [9–12]. Commercial optical tracking systems, including NDI Polaris systems and BrainLAB ExacTrac, have been widely used in medical environments, but marker ambiguity remains a recognized limitation in marker-based optical tracking [13,14]. False 3D coordinates generated by mismatches can disturb stereo reconstruction and subsequent pose estimation. Therefore, robust marker recognition and correspondence matching remain important for improving the reliability of surgical-navigation tracking systems.

To address these stereo-matching challenges, previous studies have proposed several categories of solutions. Early work primarily focused on multi-view geometric constraints. For instance, Wei et al. [15] introduced epipolar constraints for matching light-spot image points, but epipolar constraints alone may generate false points under specific viewing geometries. Yan et al. [14] developed an ambiguity-free optical tracking approach for SBRT and showed that ambiguous markers can be algorithmically reduced; however, such methods are often tied to marker-pattern correspondence and do not explicitly use contour-derived monocular depth cues. Zhang X. et al. [16] further integrated angle and distance coding for surgical-navigation stereo matching, although additional coding constraints can increase implementation complexity and computational load.

In recent years, methods based on topological patterns and projection optimization have gradually become research hotspots. Manuel Loaiza et al. [17] utilized projective invariant properties to group and uniquely identify predefined marker sets. Yan et al. [18] constructed a ghost-free reference set and employed pattern matching to detect and eliminate false points. Lin et al. [19] proposed integrating maximum contour reprojection consistency with epipolar constraints, improving matching robustness and efficiency by minimizing the 3D distance error on the surface of a virtual sphere. Wu et al. [20] and Zhang et al. [21] combined projective geometric features, spatial geometric arrangements, and motion vectors to achieve comprehensive recognition and rapid tracking of multiple instruments.

Learning-based depth estimation has explored several ways to improve the reliability of depth inference. AdaBins adapts the depth representation to individual images [22], PatchFusion combines global predictions with local refinement [23], and DCDepth progressively models low- and high-frequency depth components [24]. In related stereo and multi-view settings, edge-assisted epipolar feature aggregation [25] and parallax attention for cross-view matching [26] have been investigated. These approaches mainly address dense depth or scene reconstruction. In contrast, the present work uses the known physical size and projected contour of each spherical marker to derive a sparse metric depth prior. The prior is incorporated into an uncertainty-normalized matching cost to resolve ambiguous marker correspondences, rather than to predict a dense depth map.

Although these studies have improved marker recognition and stereo matching, robust matching remains challenging when dense or approximately coplanar markers satisfy similar epipolar constraints. The present study therefore focuses on an implementation-oriented improvement for marker-based OTS. The main novelty is not the use of epipolar geometry or ROI tracking alone, but the incorporation of contour-derived monocular depth information from the perspective deformation of spherical markers into an uncertainty-normalized global matching cost. The main contributions are as follows: (1) a contour extraction and constrained ellipse-fitting procedure is used to improve marker-center localization under high-brightness interference; (2) a matching cost is constructed by fusing epipolar residuals with monocular depth priors derived from marker contours, which helps distinguish true matches from ghost-marker candidates under coplanar ambiguity; and (3) an adaptive ROI mechanism is introduced to reduce redundant image processing while maintaining real-time tracking performance.

The remainder of this paper is organized as follows. The Method section describes the camera model, marker-detection procedure, contour-based projection-center estimation, stereo-matching cost, and ROI acceleration strategy. The Experiment section presents the experimental platform, evaluates projection-center and inter-marker distance accuracy, validates distance-dependent monocular uncertainty, compares epipolar-only, MSRC, and the proposed matching method, examines parameter sensitivity and illumination interference, and reports same-hardware runtime. The Conclusion section summarizes the findings and discusses the study limitations.

## Method

### Preliminary research

#### Camera models.

The OTS system camera model is a pinhole camera model, as shown in Fig 1. The camera coordinate system O_c_ -X_c_Y_c_Z_c_ is defined, with its origin located at the camera’s optical center. The Z_c_ axis follows the optical axis direction and is perpendicular to the imaging plane. The X_c_ and Y_c_ axes are parallel to the x-axis and y-axis of the image coordinate system.

**Fig 1:**
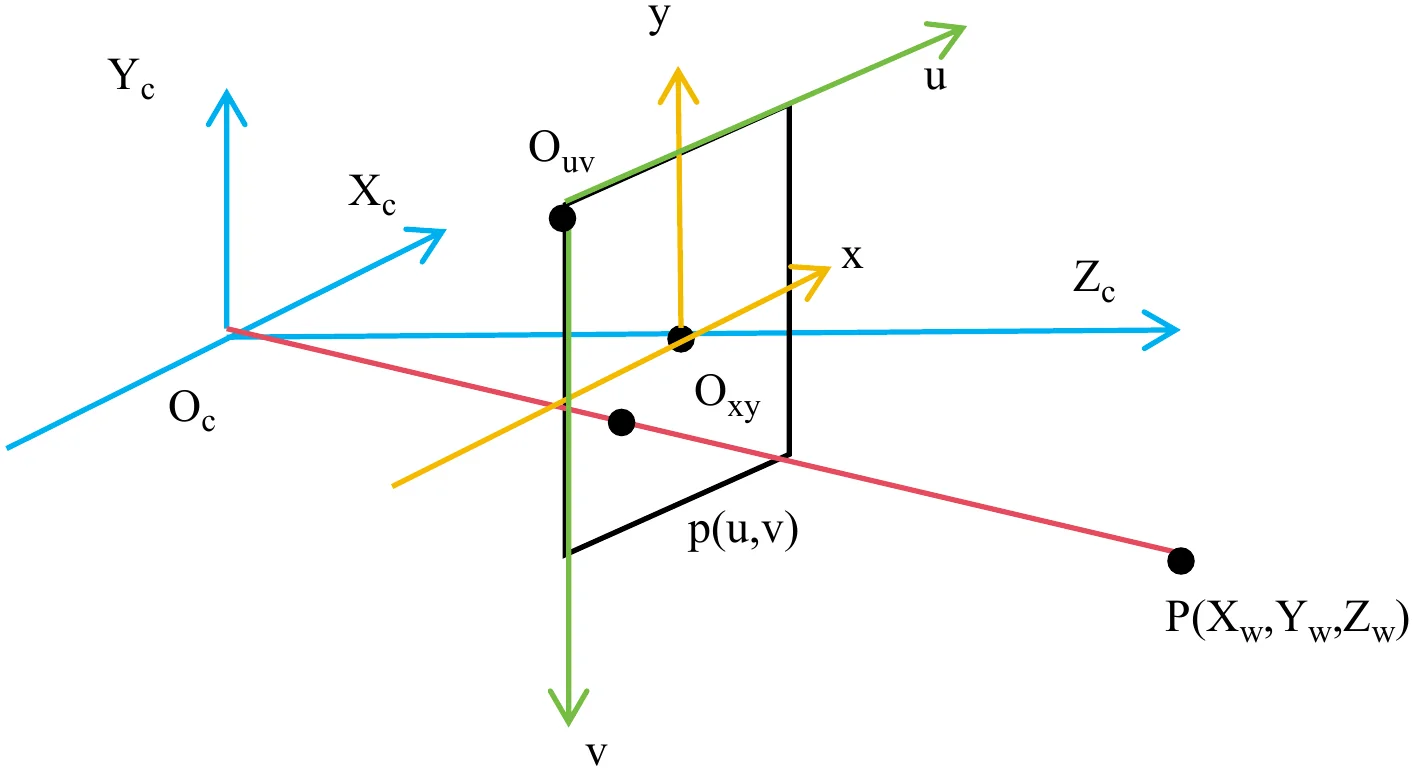
Camera model. The world, camera, and image coordinate systems and the projection of a 3D point P onto the image point p(u, v) are shown.

P = [X_W_, Y_W_, Z_W_] is the coordinate of the three-dimensional point P in the world coordinate system, and p = [u, v] is the projection of the image of point P. The relationship between a 3D point P and its projection p is expressed as:


$$\begin{array}{c} \begin{array}{c} {\text{Z}}_{\text{c}}\text{p}\text{'}=\text{K}[\text{R}|\text{t}]\text{P}\text{'} \end{array} \end{array}$$ (1)

Here, Z_c_ is a nonzero proportional factor, whose value equals the Z value of point P in the camera coordinate system. R and t are rotation matrix translational vectors, representing the conversion from the world coordinate system to the camera coordinate system, called the outer parameter. p’ and P’ are homogeneous coordinates of p and P. K is the inner parameter matrix, with the value of:


$$\begin{array}{c} \begin{array}{c} \text{K}=[\begin{array}{ccc} f_{x} & \text{0} & u_{\text{0}}\\ \text{0} & f_{y} & v_{\text{0}}\\ \text{0} & \text{0} & \text{1} \end{array}] \end{array} \end{array}$$ (2)

Here, fx and fy are the calibrated focal lengths in pixels along the u- and v-axes, respectively, and [u0, v0] is the principal point.

Each camera has several nonlinear parameters due to lens characteristics, which can cause distortion in the original image. Distortion caused by lens shape is called radial distortion. For radial distortion, quadratic and higher-order polynomial function models related to distance from the center are generally constructed to correct them, which can be described as:


$$\begin{array}{c} \begin{array}{c} \left\{ \begin{array}{c} @cx_{distorted}=x\left(\text{1}+k_{\text{1}}{\rho_{dist}}^{\text{2}}+k_{\text{2}}{\rho_{dist}}^{\text{4}}{+k}_{\text{3}}{\rho_{dist}}^{\text{6}}\right)\\ y_{distorted}=y\left(\text{1}+k_{\text{1}}{\rho_{dist}}^{\text{2}}+k_{\text{2}}{\rho_{dist}}^{\text{4}}{+k}_{\text{3}}{\rho_{dist}}^{\text{6}}\right)\\ {\rho_{dist}}^{\text{2}}=x^{\text{2}}+y^{\text{2}} \end{array}\right. \end{array} \end{array}$$ (3)

where [x,y]^T^ denotes the normalized undistorted image coordinates, [x_distorted_, y_distorted_]^T^ denotes the corresponding normalized distorted image coordinates, ρ_dist_ is the radial distance from the principal point in the normalized image plane, and k_1_, k_2_, and k_3_ are the radial distortion coefficients.

In addition to introducing radial distortion into the optical system, optical lenses also introduce tangential distortion caused by system assembly. For tangential distortion, two other parameters, p_1_ and p_2_, are generally used to correct it, as shown in equation (4):


$$\begin{array}{c} \begin{array}{c} \left\{ \begin{array}{c} x_{distorted}=x+{\text{2}p}_{\text{1}}xy+p_{\text{2}}\left({\rho_{dist}}^{\text{2}}+\text{2}x^{\text{2}}\right)\\ y_{distorted}=y+{\text{2}p}_{\text{2}}xy+p_{\text{1}}\left({\rho_{dist}}^{\text{2}}+\text{2}y^{\text{2}}\right) \end{array}\right. \end{array} \end{array}$$ (4)

Combined with the above formula, the distortion effect can be described as:


$$\begin{array}{c} \begin{array}{c} \left\{ \begin{array}{c} @rx_{distorted}=x\left(\text{1}+k_{\text{1}}{\rho_{dist}}^{\text{2}}+k_{\text{2}}{\rho_{dist}}^{\text{4}}{+k}_{\text{3}}{\rho_{dist}}^{\text{6}}\right){+\text{2}p}_{\text{1}}xy+p_{\text{2}}\left({\rho_{dist}}^{\text{2}}+\text{2}x^{\text{2}}\right)\\ y_{distorted}=y\left(\text{1}+k_{\text{1}}{\rho_{dist}}^{\text{2}}+k_{\text{2}}{\rho_{dist}}^{\text{4}}{+k}_{\text{3}}{\rho_{dist}}^{\text{6}}\right)+{\text{2}p}_{\text{2}}xy+p_{\text{1}}\left({\rho_{dist}}^{\text{2}}+\text{2}y^{\text{2}}\right) \end{array}\right. \end{array} \end{array}$$ (5)

#### Stereoscopic vision model.

The epipolar geometry of a stereo camera system is illustrated in Fig 2. Let O_l_ and O_r_ denote the optical centers of the left and right cameras, respectively, and let P be a 3D point. The plane determined by P, O_l_, and O_r_ is called the epipolar plane. Its intersections with the left and right image planes define the corresponding epipolar lines ℓ_l_ and ℓ_r._ The epipoles e_l_ and e_r_ are the projections of the opposite camera centers onto the respective image planes; equivalently, they are the intersections of the baseline with the image planes. When the optical axes are parallel, the epipoles lie at infinity rather than being absent.

**Fig 2:**
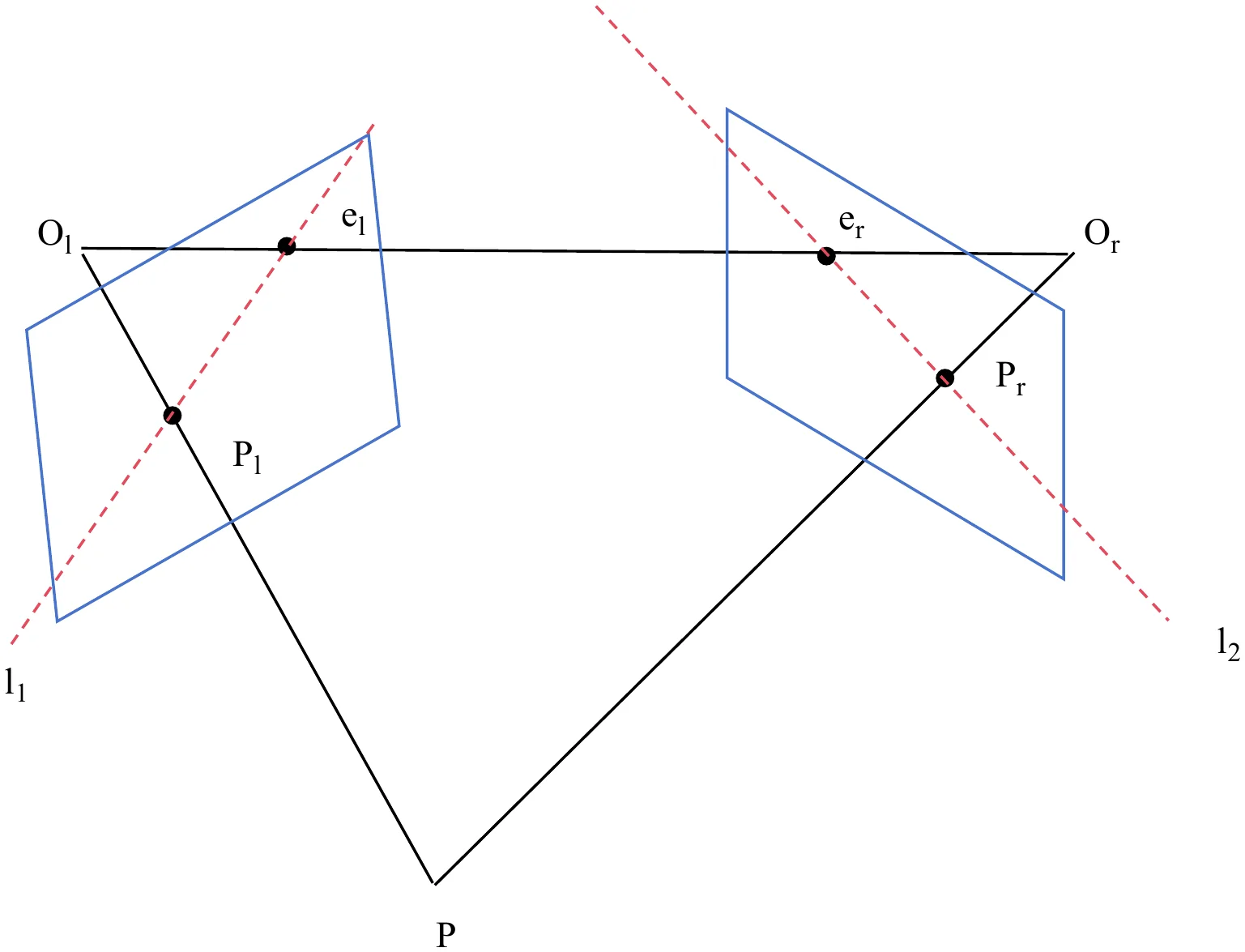
Stereo vision model. The optical centers of the left and right cameras, the baseline, the epipolar plane, and the corresponding epipolar lines used for stereo matching are shown.

Given a projection point p_l_ in the left image, its corresponding point p_r_ in the right image must lie on the associated epipolar line ℓ_r_. This constraint reduces the correspondence search from a two-dimensional image region to a one-dimensional epipolar line and thereby decreases the computational cost of stereo matching.

Once the calibration results of the stereo vision system are obtained and the correspondence between the left and right camera images is established, the coordinates of three-dimensional spatial point P can be solved using triangulation. For the left camera coordinate system o-xyz, we assume the origin of the coordinate system coincides with the origin of the world coordinate system without rotation, and the image coordinate system of the left camera is O-X_l_Y_l_, with a focal length of f_l_; The right camera coordinate system is o_r_-x_r_y_r_z_r_, corresponding image coordinate system is O_r_-X_r_Y_r_, and the focal length is fr.

Using perspective projection, the following relationships are obtained:


$$\begin{array}{c} \begin{array}{c} \left\{ \begin{array}{c} {\text{s}}_{\text{l}}[\begin{array}{c} {\text{X}}_{\text{l}}\\ {\text{Y}}_{\text{l}}\\ \text{1} \end{array}]=[\begin{array}{ccc} {\text{f}}_{\text{l}} & \text{0} & \text{0}\\ \text{0} & {\text{f}}_{\text{l}} & \text{0}\\ \text{0} & \text{0} & \text{1} \end{array}][\begin{array}{c} \text{x}\\ \text{y}\\ \text{z} \end{array}]\\ {\text{s}}_{\text{r}}[\begin{array}{c} {\text{X}}_{\text{r}}\\ {\text{Y}}_{\text{r}}\\ \text{1} \end{array}]=[\begin{array}{ccc} {\text{f}}_{\text{r}} & \text{0} & \text{0}\\ \text{0} & {\text{f}}_{\text{r}} & \text{0}\\ \text{0} & \text{0} & \text{1} \end{array}][\begin{array}{c} {\text{x}}_{\text{r}}\\ {\text{y}}_{\text{r}}\\ {\text{z}}_{\text{r}} \end{array}] \end{array}\right. \end{array} \end{array}$$ (6)

The positional relationship between the left and right cameras can be expressed using the spatial transformation matrix M_lr_


$$\begin{array}{c} \begin{array}{c} \left[\begin{array}{c} {\text{x}}_{\text{r}}\\ {\text{y}}_{\text{r}}\\ {\text{z}}_{\text{r}} \end{array}]={\text{M}}_{\text{lr}}[\begin{array}{c} \text{x}\\ \text{y}\\ \text{z}\\ \text{1} \end{array}]=[\begin{array}{cccc} {\text{r}}_{\text{1}} & {\text{r}}_{\text{2}} & {\text{r}}_{\text{3}} & {\text{t}}_{\text{x}}\\ {\text{r}}_{\text{4}} & {\text{r}}_{\text{5}} & {\text{r}}_{\text{6}} & {\text{t}}_{\text{y}}\\ {\text{r}}_{\text{7}} & {\text{r}}_{\text{8}} & {\text{r}}_{\text{9}} & {\text{t}}_{\text{z}} \end{array}][\begin{array}{c} \text{x}\\ \text{y}\\ \text{z}\\ \text{1} \end{array}\right] \end{array} \end{array}$$ (7)

After deduction:


$$\begin{array}{c} \begin{array}{c} \left\{ \begin{array}{c} @cx=zX_{l}/f_{l}\\ y=zY_{l}/f_{l}\\ z=\frac{f_{l}\left(f_{r}t_{x}-X_{r}t_{z}\right)}{X_{r}\left(r_{\text{7}}X_{l}+r_{\text{8}}Y_{l}+f_{l}r_{\text{9}}\right)-f_{r}\left(r_{\text{1}}X_{l}+r_{\text{2}}Y_{l}+f_{l}r_{\text{3}}\right)} \end{array}\right. \end{array} \end{array}$$ (8)

### Mark detection

In optical positioning systems, the accuracy of pixel coordinates extraction at the center of markers directly affects the system’s operational accuracy. The core lies in identifying and segmenting markers in the image and calculating the position of the center of mass. Due to the reflective coating on the surface of the marks, these marks reflect near-infrared light emitted from the optical probe back to the camera, forming nearly round, high-brightness spots. However, most of the area around the mark is nearly completely black, forming a sharp contrast with the marker.

#### Contour extraction.

However, the high intensity of reflection on the surfaces of other medical instruments in the operating room can cause localized highlights, seriously interfering with the extraction of marker contours. In addition, uneven lighting easily causes fractures at the edges extracted by traditional binarization methods. To this end, this chapter adopts a boundary evolution method combining edge detection and conditional dilation to achieve precise extraction of marker profiles.

First, the Canny algorithm is employed to extract high-frequency edges from the image, thereby obtaining the initial boundary set E_edges_. Since the boundaries at this stage typically manifest as unclosed hollow line structures, the direct detection of external topological contours is unfeasible. To constrain the valid boundaries for geometric evolution and suppress background reflections, a fixed threshold T_bright_ is applied to binarize the grayscale image, constructing a mask of high-luminance candidate regions, denoted as M_bright_. Subsequently, utilizing E_edges_ as the seed points for growth, an elliptical structuring element B is introduced to perform iterative morphological filling under the topological constraints of the mask M_bright_. The mathematical evolution equation for the k-th iteration is given by:


$$\begin{array}{c} \begin{array}{c} X_{k+\text{1}}=\left(X_{k}⨁B\right)\cap {\text{M}}_{\text{bright}}\cup {\text{E}}_{\text{edges}} \end{array} \end{array}$$ (9)

where ⊕ denotes the standard morphological dilation operator, and ∩ and ∪ represent the matrix bitwise AND and OR operations, respectively, with the initial state defined as X_0_ = E_edges_. During this process, the dilation operator guides the boundaries to fill the interior of the target, while the intersection operation with M_bright_ acts as a barrier, strictly preventing the features from propagating into the background reflection regions. The iteration terminates when the convergence condition X_k+1_ = X_k_ is satisfied, outputting the solid marker connected component X_final_ = X_k+1_.

Based on X_final_, the contour detection function in OpenCV is utilized to extract the set of external closed contours C^d^={c_i_^d^}, where c_i_^d^={p_j_^d^} and p_j_^d^ represents an individual pixel within the i-th contour. Because performing distortion correction on the entire image is highly time-consuming, this study applies the correction exclusively to each contour c_i_^d^ obtained via contour detection to enhance real-time performance. Ultimately, the corrected contour c_i_= {p_j_} and the corrected contour set C={c_i_} are obtained.

After the closed candidate contours are extracted, radial-consistency screening is applied to reject irregular reflection regions. For each contour c_i_, the normalized dispersion of the radial distances from its contour points to the contour centroid is evaluated. A smaller dispersion indicates greater radial consistency. To avoid rejecting valid marker contours distorted by perspective projection, moderate ellipticity is permitted. Specifically, let d_j_ denote the distance from a contour point p_j_ to the centroid oi, and let d̄ᵢ denote the mean radial distance of contour c_i_. For a contour containing M_i_ points, the normalized radial-distance RMSE is expressed as Eq. (10). A candidate contour is retained only when RMSE_i_ ≤ T_r_; otherwise, it is rejected as an irregular reflection region. Based on the parameter-sensitivity experiment described in Parameter sensitivity analysis, T_r_ was set to 0.06 in all subsequent experiments.


$$\begin{array}{c} \begin{array}{c} {\text{RMSE}}_{\text{i}}=\sqrt{\frac{\text{1}}{M_{i}}\sum_{j=\text{1}}^{M_{i}}{\left[\left(d_{j}-{\overset{―}{d}}_{i}\right)/{\overset{―}{d}}_{i}\right]}^{\text{2}}} \end{array} \end{array}$$ (10)

The second filtering criterion constrains the area enclosed by each candidate contour. Preliminary experiments were conducted to characterize the projected marker area at different working distances. The maximum enclosed area observed at 0.4m was 1850 pixels, whereas the minimum area observed at 1.5m was 90 pixels. Therefore, only candidate contours with an enclosed area between 90 and 1850 pixels are retained.

To further suppress closed contours caused by reflections from metallic instruments, the mean grayscale intensity within each candidate contour is evaluated using the original grayscale image. Let Ωᵢ denote the set of pixels enclosed by contour cᵢ and Nᵢ denote the number of pixels in Ωᵢ. The mean grayscale intensity is calculated as


$$\begin{array}{c} \begin{array}{c} I_{i}=\frac{\text{1}}{N_{i}}\sum_{p\in \Omega_{i}}I(p) \end{array} \end{array}$$ (11)

Under the imaging conditions used in this study, the marker spheres produce stronger and more spatially extensive near-infrared reflections than metallic surfaces. Therefore, a candidate contour is retained only when its mean grayscale intensity satisfies (Ī_i_ > T_I_), where T_I_ is an empirically determined intensity threshold. A contour is finally classified as a marker candidate only if it satisfies the radial-consistency, enclosed-area, and mean-intensity criteria.

Up to this point, the system is capable of robustly and accurately extracting target markers from a single-frame image even in the presence of high-intensity reflection interference. However, optical tracking systems typically require high-frequency, real-time pose estimation within continuous video streams. If full-frame blind searching and feature filtering were performed on every frame of the video stream, the system’s computational capacity would be overwhelmed, leading to a severe degradation in the frame rate. To address the dynamic tracking requirements in continuous video streams, this paper adopts an adaptive Region of Interest (ROI) cascade extraction mechanism that combines down-sampled detection with original-resolution local refinement. The procedure consists of two stages: global coarse screening and local precise extraction.

Global Down-sampled Detection Stage: The system introduces a periodic scanning mechanism, compressing the original high-resolution image to a scale space of 1/n every N_scan_ frames. A full-field rapid scan is executed on the down-sampled image to initially identify newly appearing markers within the field of view. By leveraging the spatiotemporal continuity of the images, this step obtains the rough positions of the markers, providing candidate centers for subsequent processing.

Adaptive ROI Local Precise Extraction Stage: As illustrated in Fig 3, based on the acquired candidate centers, the system dynamically calculates and constructs adaptive ROI boundaries by integrating the projection radius of the markers on the image and the predefined maximum inter-frame physical displacement. Subsequently, the system traces back to the original high-resolution video frame, performing feature extraction and contour fitting exclusively within the local neighborhood defined by the ROI. This completes the alternating tracking pipeline from low-resolution global screening to high-resolution local processing.

**Fig 3:**
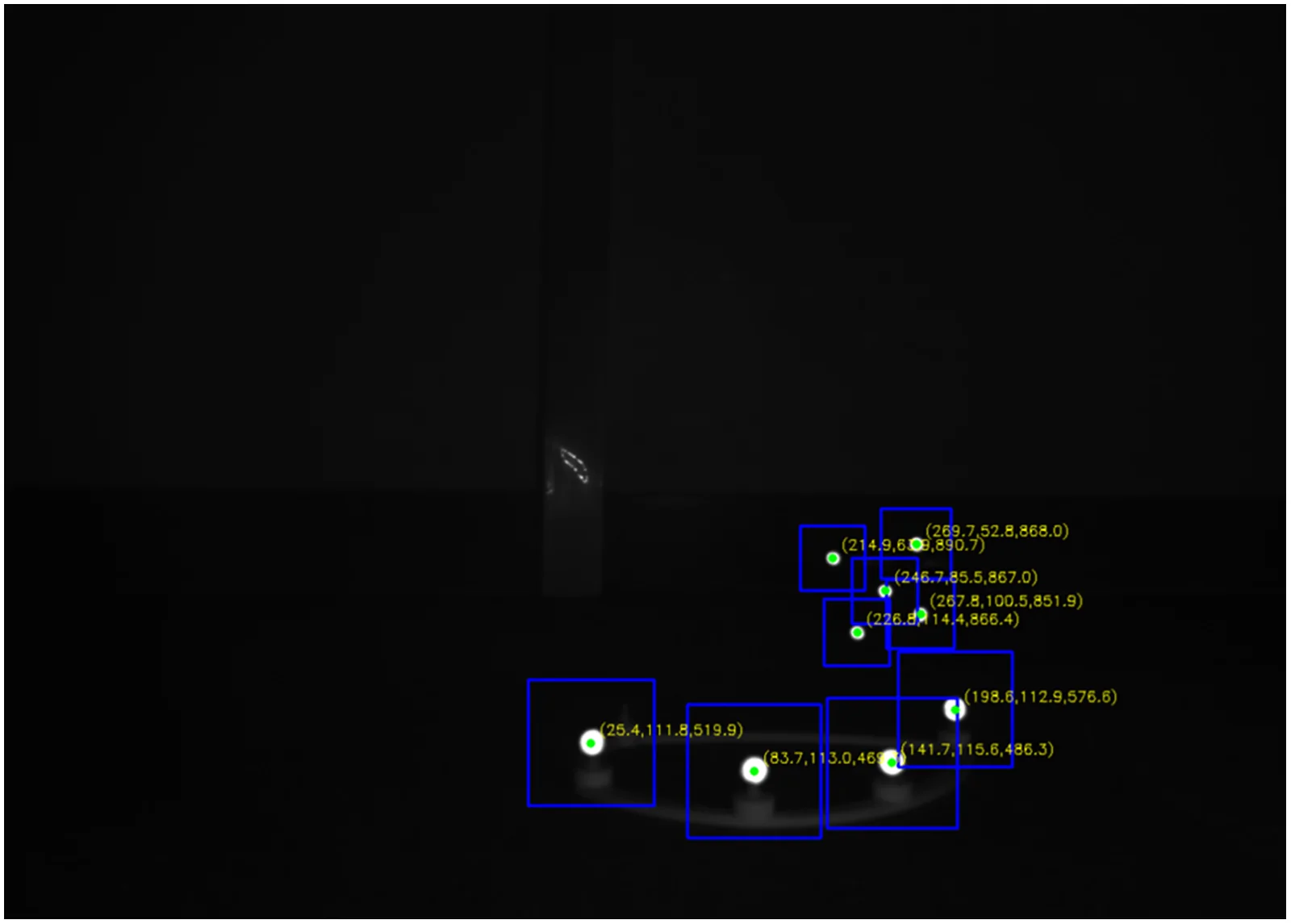
Adaptive ROI extraction. Candidate marker centers detected in the down-sampled image are used to define local regions of interest for high-resolution contour extraction and refinement.

#### Calculation of projection centers.

As shown in Fig 4, the perspective projection of a spherical target produces an elliptical image contour. The center of this ellipse does not generally coincide with the image projection of the three-dimensional sphere center. Nevertheless, owing to the symmetry of sphere projection, the camera principal point, the ellipse center, and the major-axis direction are collinear in the normalized image plane. Based on this geometric property, a four-parameter constrained ellipse model is introduced to estimate the ellipse parameters, followed by a perspective correction to recover the projected sphere center.

**Fig 4:**
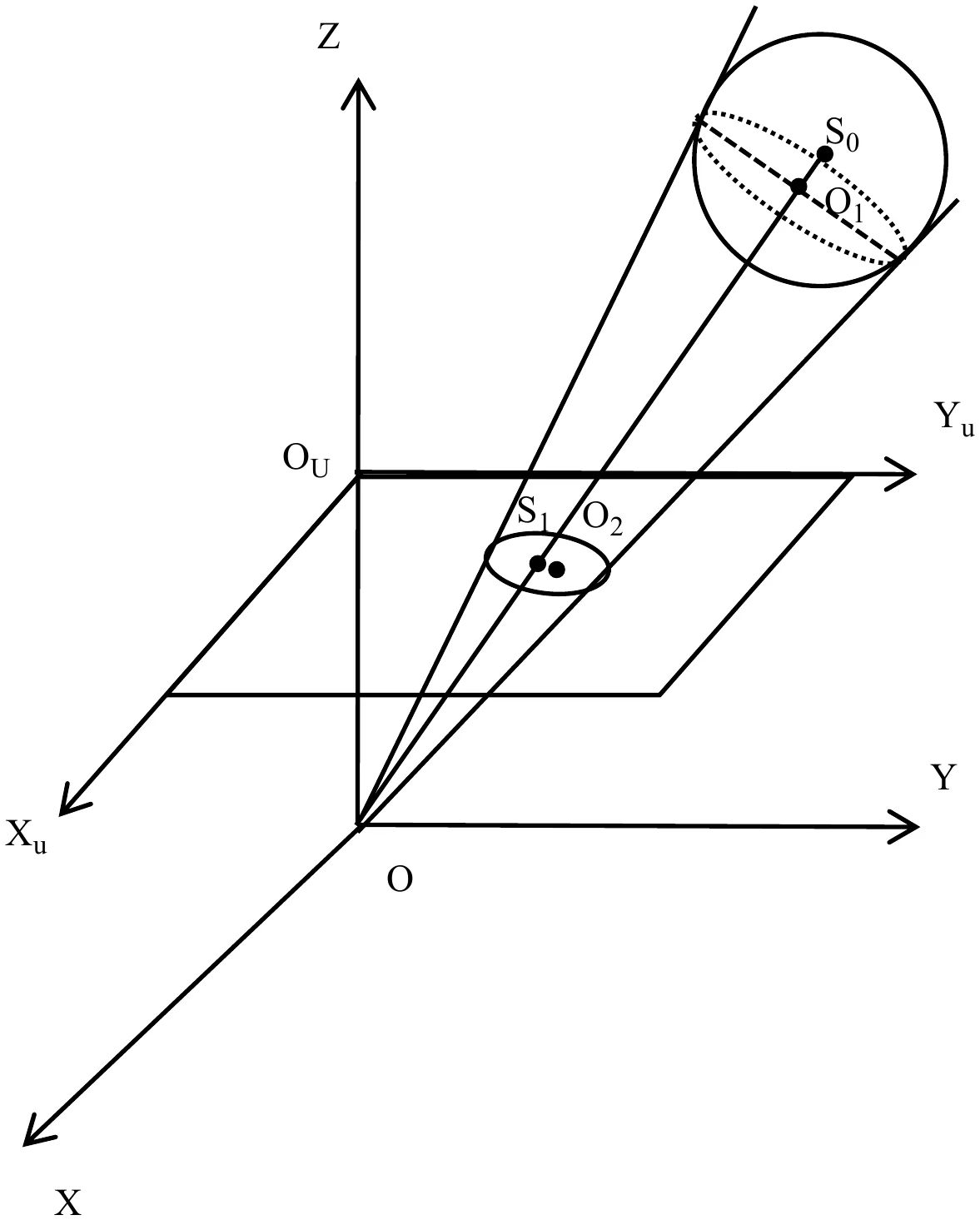
Space spherical imaging model. The projection of a spherical marker forms an ellipse on the image plane, and the relationship between the projected ellipse and the sphere-center projection is used for projection-center estimation.

**Four-parameter ellipse model**: Let (u_i_,v_i_) be the distortion-corrected pixel coordinates of the i-th contour point. Given the focal lengths (f_x_,f_y_) and principal point (u_0_,v_0_), the normalized coordinates are


$$\begin{array}{c} \begin{array}{c} x_{i}=\frac{u_{i}-u_{\text{0}}}{f_{x}},\;y_{i}=\frac{v_{i}-v_{\text{0}}}{f_{y}}. \end{array} \end{array}$$ (12)

The unit vectors along the major and minor axes are defined as


$$\begin{array}{c} q(\theta )=\left[\begin{array}{c} @l@cos\theta \\ sin\theta \end{array}\right],q_{⊥}(\theta )=\left[\begin{array}{c} @l@-sin\theta \\ cos\theta \end{array}\right]. \end{array}$$ (13)

The ellipse center is parameterized as


$$\begin{array}{c} \begin{array}{c} c_{e}=\rho \text{q}(\theta ),\;\rho \geq \text{0}. \end{array} \end{array}$$ (14)

where (ρ) is the normalized distance from the principal point to the ellipse center. Because the center direction and the major-axis direction share the same parameter θ, Eq. (14) strictly enforces the collinearity of the principal point, ellipse center, and ellipse major axis.

To guarantee valid semi-axis lengths, the major and minor semi-axes are parameterized as


$$\begin{array}{c} \begin{array}{c} b=\text{exp}(\beta ),\;\;a=b\text{exp}(\gamma ),\;\;\gamma \geq \text{0}. \end{array} \end{array}$$ (15)

Therefore, (a ≥ b > 0) is always satisfied. The unknown parameter vector is


$$\begin{array}{c} \begin{array}{c} \omega =[\begin{array}{cccc} \rho & \theta & \beta & \gamma \end{array}]\text{T}. \end{array} \end{array}$$ (16)

A general ellipse has five geometric degrees of freedom. The proposed collinearity constraint couples its center position with its orientation and reduces the number of independent parameters from five to four.

**Ellipse residual:** For a normalized contour point p_i_=(x_i_,y_i_)^T^ its coordinates in the local ellipse frame are


$$\begin{array}{c} \begin{array}{c} \left\{ \begin{array}{c} @l\xi_{i}=x_{i}cos\theta +y_{i}sin\theta -\rho \\ \eta_{i}={-x}_{i}sin\theta +y_{i}cos\theta \end{array}\right. \end{array} \end{array}$$ (17)

The constrained ellipse equation is


$$\begin{array}{c} \begin{array}{c} g_{i}(\omega )=\frac{\xi_{i}^{\text{2}}}{a^{\text{2}}}+\frac{\eta_{i}^{\text{2}}}{b^{\text{2}}}-\text{1}. \end{array} \end{array}$$ (18)

Because(a ≥ b > 0), the resulting quadratic form is always positive definite. Hence, the fitted curve is guaranteed to be a real, non-degenerate ellipse rather than another type of conic.

Instead of directly minimizing the algebraic residual gi, an approximate geometric residual is used:


$$\begin{array}{c} \begin{array}{c} e_{i}(\omega )=f_{eff}\frac{g_{i}(\omega )}{{\left\|\nabla g_{i}(\omega )\right\|}_{\text{2}}+\varepsilon },\;\;f_{eff}=\frac{f_{x}+f_{y}}{\text{2}}, \end{array} \end{array}$$ (19)

where ε is a small positive constant and f_eff_ = (f_x_ + f_y_)/2 is the effective focal length in pixels. The residual e_i_ approximates the signed normal distance from the contour point to the ellipse and is expressed approximately in pixels.

**Robust optimization and initialization**:The ellipse parameters are obtained by minimizing a Huber robust objective:


$$\begin{array}{c} \begin{array}{c} \omega^{*}=\text{arg}\underset{\omega }{\text{min}}\sum_{i=\text{1}}^{N}{\text{L}}_{\delta }\left(e_{i}(\omega )\right), \end{array} \end{array}$$ (20)

where L_δ_ is the Huber loss and δ = 0.5 pixel in the implementation. The Huber loss behaves quadratically for small residuals and linearly for large residuals, thereby reducing the influence of local edge irregularities.

Because Eq. (20) is a nonlinear optimization problem, cv2.fitEllipse is first used to estimate an approximate center and axis lengths. Its output is used only for initialization. In particular, the unconstrained orientation returned by cv2.fitEllipse is discarded. If the normalized initial center is (x_c0_,y_c0_), the initial constrained parameters are calculated as


$$\begin{array}{c} \begin{array}{c} \rho_{\text{0}}=\sqrt{x_{c\text{0}}^{\text{2}}+y_{c\text{0}}^{\text{2}}},\;\;\theta_{\text{0}}=\text{atan}\text{2}\left(y_{c\text{0}},x_{c\text{0}}\right), \end{array} \end{array}$$ (21)

$$\begin{array}{c} \begin{array}{c} \beta_{\text{0}}={\text{ln}b}_{\text{0}},\;\;\gamma_{\text{0}}=\text{max}\left(\text{0},\text{ln}\frac{a_{\text{0}}}{b_{\text{0}}}\right). \end{array} \end{array}$$ (22)

Thus, the initial ellipse already satisfies the proposed collinearity constraint. During optimization, all four parameters are updated jointly. Consequently, the ellipse center is continuously refined according to


$$\begin{array}{c} \begin{array}{c} c_{e}^{\left(k\right)}=\rho^{\left(k\right)}[\begin{array}{c} {\text{cos}\theta }^{\left(k\right)}\\ {\text{sin}\theta }^{\left(k\right)} \end{array}], \end{array} \end{array}$$ (23)

while the center direction and the major-axis direction remain collinear throughout the optimization. The problem is solved using the trust-region reflective nonlinear least-squares algorithm.

**Fixed-threshold outlier rejection**: After the first robust fit, the geometric residual of each contour point is evaluated. A fixed threshold of


$$\begin{array}{c} \begin{array}{c} T_{f}=\text{1}.\text{0}\text{pixel} \end{array} \end{array}$$ (24)

is used for outlier rejection. The inlier set is defined as


$$\begin{array}{c} \begin{array}{c} I=\left\{i:\left|e_{i}\right|\leq T_{f}\right\} \end{array} \end{array}$$ (25)

Points with (|ei| > T_f_) are removed. If at least five contour points remain, the ellipse is fitted again using only the inliers, with the first fitting result used as the initial estimate. Finally, the optimized ellipse is transformed from the normalized image plane back to the pixel coordinate system.

**Sphere-center projection correction**: As shown in Fig 4, under perspective projection, the center of the fitted ellipse does not exactly coincide with the image projection of the three-dimensional sphere center. Therefore, a subsequent correction is applied along the radial direction from the camera principal point.

Let h be the pixel distance from the principal point to the fitted ellipse center, a_p_ the major semi-axis in pixels, and f_eff_ the effective focal length in pixels. The corrected radial distance is calculated as


$$\begin{array}{c} \begin{array}{c} l=f_{eff}\text{tan}\left[\frac{\text{1}}{\text{2}}\left(\text{arctan}\frac{h+a_{p}}{f_{eff}}+\text{arctan}\frac{h-a_{p}}{f_{eff}}\right)\right] \end{array} \end{array}$$ (26)

Let o_p_ and c_p_ denote the principal point and fitted ellipse center, respectively. The corrected sphere-center projection is


$$\begin{array}{c} \begin{array}{c} {\text{s}}_{p}={\text{o}}_{p}+l\frac{{\text{c}}_{p}-{\text{o}}_{p}}{{\left\|{\text{c}}_{p}-{\text{o}}_{p}\right\|}_{\text{2}}} \end{array} \end{array}$$ (27)

The corrected sphere-center projection is subsequently used for stereo correspondence, triangulation, and three-dimensional reconstruction.

### Precise stereo matching

In Marker-based Optical Tracking Systems (OTS), the matching of multi-view 2D feature points is a prerequisite for 3D reconstruction, with the epipolar constraint serving as the core geometric criterion. Under the perspective projection model, a projection point p_l_ on the first camera’s image plane intrinsically corresponds to an epipolar line on the second camera’s image plane. Mathematically, this relationship is defined by the fundamental matrix F, and the corresponding projection point p_r_ strictly satisfies the following epipolar geometry equation:


$$\begin{array}{c} \begin{array}{c} p_{\text{r}}^{\text{T}}Fp_{\text{l}}=\text{0} \end{array} \end{array}$$ (28)

However, the epipolar constraint exhibits significant underlying limitations when processing marker sets with high density or specific topological structures. When a large number of densely distributed markers are present within the tracking field of view, or when the markers on a target rigid body exhibit a coplanar or approximately coplanar arrangement, multiple candidate markers’ 2D projections in the second camera’s view often simultaneously fall onto (or close to) the matching tolerance range of the same epipolar line l2. As illustrated in Fig 5, assume there are two real physical markers, P and Q, within the system’s field of view, generating projection point pairs (P1, P2) and (Q1, Q2) on the image planes of the left and right cameras (with optical centers O1 and O2), respectively. Under ideal epipolar matching, homologous projection rays (e.g., O1P1 and O2P2 will precisely intersect in space, thereby reconstructing the real point P through triangulation.

**Fig 5:**
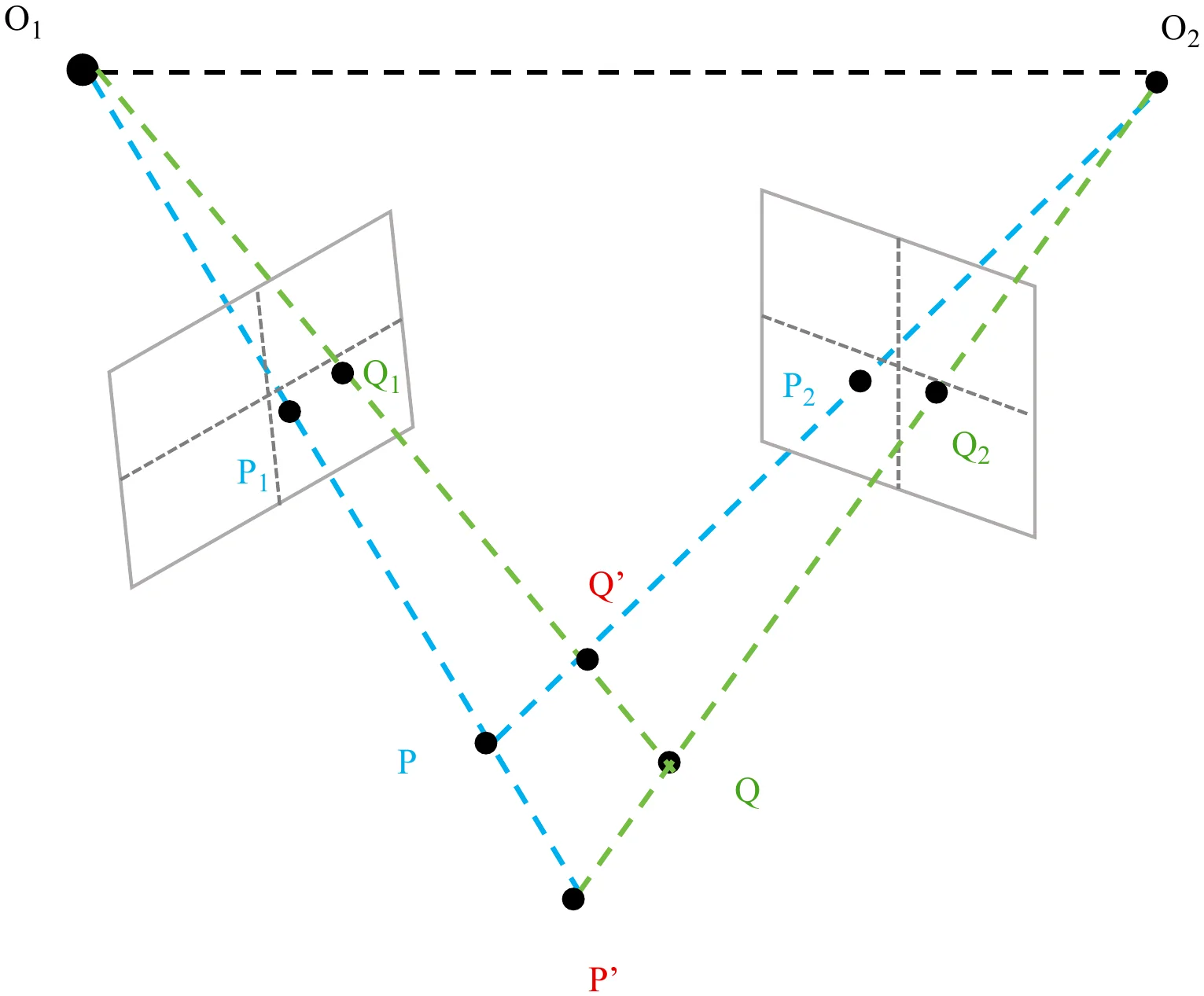
Schematic diagram of ghost point mismatches. Under coplanar epipolar geometry, non-homologous projection rays can be incorrectly paired, producing false reconstructed points in 3D space.

Nevertheless, when target markers present a specific topological structure in space (for instance, when points P, Q and the baseline connecting the optical centers O_1_、O_2_ are approximately coplanar), multiple corresponding projection rays O_1_P_1_, O_1_Q_1_, O_2_P_2_, O_2_Q_2_) will fall into the same epipolar plane. In this scenario, the uniqueness of the epipolar constraint completely fails: the projection ray O_1_P_1_ from the left camera intersects not only with the true homologous ray O_2_P_2_ from the right camera, but also erroneously intersects with the non-homologous ray O_2_Q_2_ in space. This stereo matching ambiguity, triggered by epipolar coplanarity, leads the matching algorithm to generate false correlations. Once the system incorrectly associates non-homologous projections (such as P_1_ with Q_2_, or Q_1_ with P_2_) and performs triangulation, false coordinate nodes Q’ and P’ (as indicated by the red nodes in the figure) will be computed at locations in physical space that are actually empty. These false 3D coordinates generated by mismatches are defined as “ghost points.” They not only consume the system’s computational resources but also severely disrupt the subsequent registration and pose estimation of the target rigid body point cloud.

To reduce matching ambiguity induced by epipolar coplanarity and high-density marker configurations, point-based multi-view geometry can be supplemented with independent contour information. This paper therefore proposes a matching strategy based on multi-dimensional feature fusion. Beyond traditional centroid-based epipolar matching, the method uses topological and morphological information from the 2D marker contour for monocular depth estimation. Because physical spherical markers exhibit perspective deformation under different distances and viewpoints, the monocular analysis provides an additional depth-related prior for each candidate match. By combining this prior with the epipolar residual in a global matching cost, the proposed method increases the separation between true matches and false correlations in the tested ambiguity scenarios.

#### Epipolar line error.

Assuming the Optical Tracking System (OTS) has achieved high-precision calibration, the precise fundamental matrix F can be obtained in advance. For a feature point p_l_ extracted from the left view, its corresponding epipolar line equation in the right view can be directly derived as ℓ_r_ = F p_l_. However, constrained by the inherent errors during the measurement of the marker’s centroid, the corresponding true matching point p_r_ rarely falls absolutely precisely on the epipolar line ℓ_r_; instead, it maintains a minuscule deviation from it. If p_r_’ is defined as the orthogonal projection of p_r_ onto the epipolar line ℓ_r_, the perpendicular distance between them can be expressed as dist(p_r_,ℓ_r_) = dist(p_r_’,p_r_).

In practical matching, a global fixed threshold T_ep_ is used to construct the candidate correspondence set. For a feature point p_l_ in the left image, a point p_r_ in the right image is retained as a candidate only if


$$\begin{array}{c} \begin{array}{c} \text{dist}({\text{p}}_{\text{r}},{\text{𝓁}}_{\text{r}})\leq {\text{T}}_{\text{ep}} \end{array} \end{array}$$ (29)

This threshold is used exclusively for candidate screening and is distinct from the epipolar uncertainty scale used in the subsequent matching-cost calculation.

#### Monocular estimate.

Under the perspective projection model, a spatial sphere and the camera’s optical center (center of projection) constitute a right circular cone. The intersection of this tangent cone with the image plane yields the observed ellipse. First, utilizing the given ellipse parameters (center coordinates, major and minor axes, and rotation angle), a homogeneous conic matrix C_p_ is constructed within the pixel coordinate system. This matrix satisfies the algebraic equation for any homogeneous pixel coordinate p=[u,v,1]^T^ lying on the ellipse boundary:


$$\begin{array}{c} \begin{array}{c} p^{\text{T}}C_{p}p=\text{0} \end{array} \end{array}$$ (30)

There is affine distortion with scale and principal offset in the pixel coordinate system, which must be transformed into a dimensionless normalized image coordinate system using the camera’s internal parameter matrix K. By transforming p = Kx through inverse perspective and substituting it into Eq. (30), we get:


$$\begin{array}{c} \begin{array}{c} (Kx{)}^{\text{T}}C_{p}(Kx)=x^{\text{T}}Qx \end{array} \end{array}$$ (31)

Among them:


$$\begin{array}{c} \begin{array}{c} {\text{Q}=\text{K}}^{\text{T}}{\text{C}}_{\text{p}}\text{K} \end{array} \end{array}$$ (32)

As illustrated in Fig 6, for a spherical marker centered at the 3D coordinate c, the marker diameter was 11.5 mm; therefore, the physical radius used in the monocular reconstruction was r_m_ = 5.75 mm. The angle between any tangent ray vector x and the sphere-center vector c therefore satisfies:

**Fig 6:**
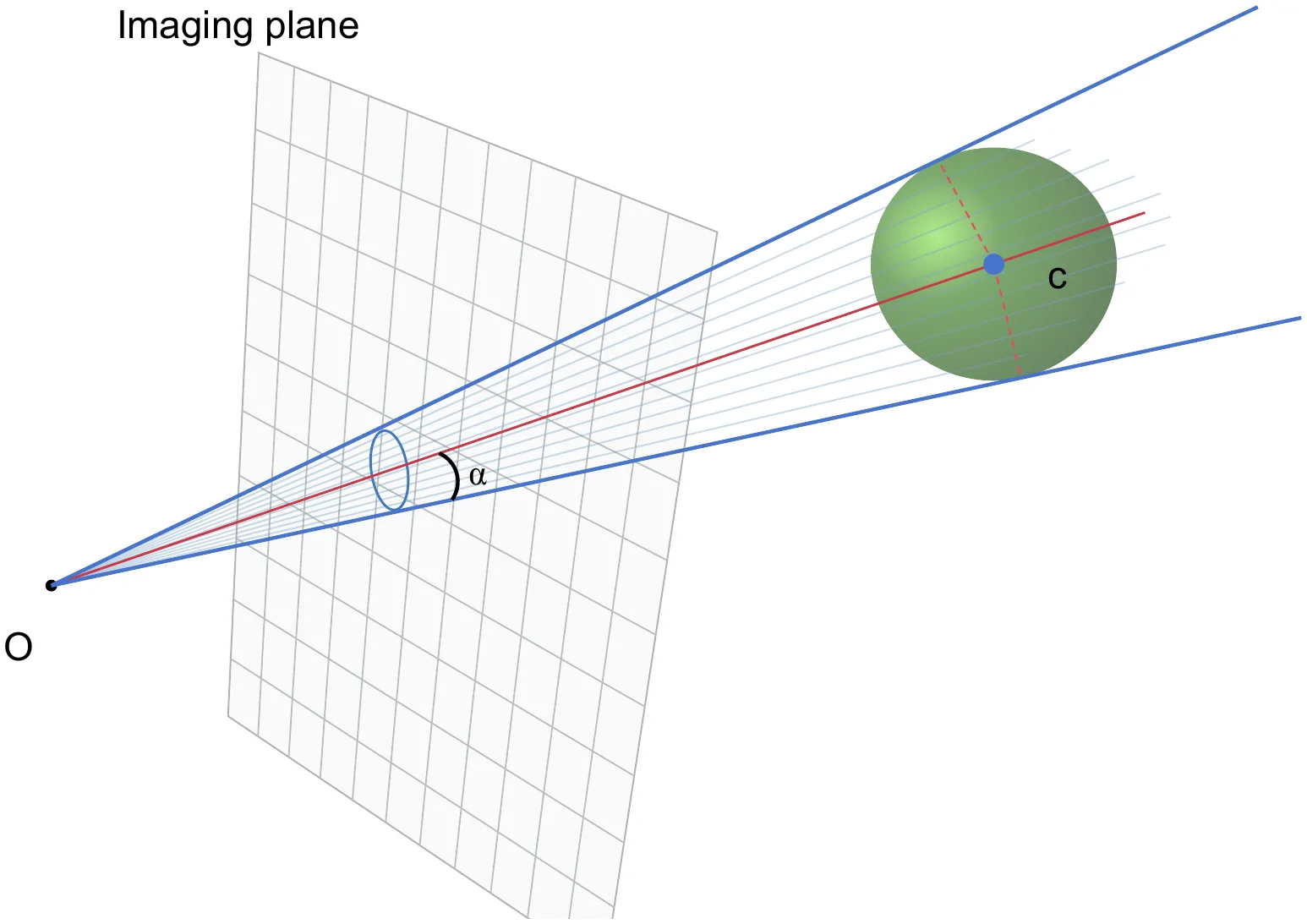
Marker projection model. The camera center, tangent cone, imaging plane, and projected ellipse of the spherical marker are used to derive the contour-based monocular depth prior.


$$\begin{array}{c} \begin{array}{c} sin(\alpha )=\frac{r_{m}}{\left\|c\right\|} \end{array} \end{array}$$ (33)

Based on the vector dot product formula c^T^x = ‖c‖‖x‖cos(α)and Eq. (33), the tangent cone equation under pure geometry can be derived as follows:


$$\begin{array}{c} \begin{array}{c} {\left({\text{c}}^{\text{T}}\text{x}\right)}^{\text{2}}=\left({\left\|\text{c}\right\|}^{\text{2}}-{r_{m}}^{\text{2}}\right){\left\|\text{x}\right\|}^{\text{2}} \end{array} \end{array}$$ (34)

This can be simplified into a matrix quadratic form:


$$\begin{array}{c} \begin{array}{c} x^{\text{T}}\left[\text{c}c^{\text{T}}-\left({\left\|\text{c}\right\|}^{\text{2}}-{r_{m}}^{\text{2}}\right)\text{I}\right]x=\text{0} \end{array} \end{array}$$ (35)

This implies that the matrix Q computed from the image is equal to this theoretical geometric matrix, up to a non-zero scalar factor κ:


$$\begin{array}{c} \begin{array}{c} \text{Q}=\kappa \left[\text{c}c^{\text{T}}-\left({\left\|\text{c}\right\|}^{\text{2}}-{r_{m}}^{\text{2}}\right)\text{I}\right] \end{array} \end{array}$$ (36)

Performing an eigendecomposition on the symmetric matrix Q yields.The uniquely signed eigenvalue λs = κr_m_^2^: its corresponding eigenvector represents the direction of the symmetry axis of the right circular cone, i.e., the unit direction vector pointing from the camera’s optical center to the 3D sphere center.Any vector v within the plane perpendicular to the sphere center direction is also an eigenvector, constituting an eigenspace of multiplicity two with a repeated eigenvalue of λp = -κ(D^2^-r_m_^2^).

By leveraging the proportional relationship between the eigenvalues of the tangent cone matrix, the Euclidean distance D from the optical center to the sphere center can be analytically derived as follows:


$$\begin{array}{c} \begin{array}{c} D=r_{m}\sqrt{\frac{\lambda_{s}-\lambda_{p}}{\lambda_{s}}} \end{array} \end{array}$$ (37)

At this point, the scalar distance D is known, and the unit direction vector is precisely the normalized eigenvector $v_{s}$ corresponding to the eigenvalue λ_s_. Consequently, the absolute 3D coordinate c of the sphere within the camera coordinate system can be accurately reconstructed as:


$$\begin{array}{c} \begin{array}{c} c=D\cdot v_{s} \end{array} \end{array}$$ (38)

The Euclidean distance between the 3D coordinates derived from monocular estimation and those from binocular triangulation is calculated as follows:


$$\begin{array}{c} \begin{array}{c} E^{sm}=\left\|c^{s}-c^{m}\right\| \end{array} \end{array}$$ (39)

#### Construct a matching cost function.

To improve marker matching under ambiguous imaging configurations, an uncertainty-normalized cost function is constructed by combining the epipolar residual with the contour-derived monocular depth residual. In the implemented algorithm, the epipolar and monocular terms use different uncertainty models.

Preliminary measurements showed that variations in the epipolar residual caused by contour-point statistics were small compared with the error associated with stereo-camera calibration. Consequently, the contour-perimeter-dependent epipolar uncertainty was not evaluated separately for each marker. Instead, a fixed epipolar normalization scale was used throughout the reported experiments:


$$\begin{array}{c} \begin{array}{c} \sigma_{\text{ep}}=\text{0}.\text{4px} \end{array} \end{array}$$ (40)

The value σ_ep_ is used only to normalize the epipolar residual in the matching cost, whereas the fixed threshold T_ep_ defined above is used only to generate candidate correspondences. Thus, T_ep_ and σ_ep_ serve different purposes and are not interchangeable.

The accuracy of visual localization is intrinsically limited by the camera’s imaging geometry. Based on the pinhole camera model, the projected marker radius r_img_ (pixels) and camera-to-marker distance D (mm) satisfy r_img_ = f_eff_·r_m_/D. By applying a first-order Taylor expansion, the anisotropic uncertainty of the monocular depth estimation can be derived.


$$\begin{array}{c} \begin{array}{c} \sigma_{mono}\left(\text{D}\right)\approx \delta_{\text{init}}+\frac{{\text{D}}^{\text{2}}}{f_{\text{eff}}\cdot r_{m}}\cdot \delta_{\text{p}} \end{array} \end{array}$$ (41)

Here, f_eff_ is the effective focal length in pixels, r_m_ is the known physical radius of the spherical marker, and δₚ is the contour-localization uncertainty in the image plane. The constant δ_init_ represents the distance-independent baseline uncertainty associated with residual camera-calibration error, model mismatch, and other error sources not captured by the first-order propagation of pixel noise. In the reported experiments, δ_init_ was empirically set to 5 mm based on preliminary measurements.

Unlike the fixed epipolar normalization scale, the monocular depth uncertainty σ_mono_(D) is evaluated for each candidate match according to its estimated camera-to-marker distance D. Because σ_mono_(D) increases approximately quadratically with distance, the contribution of the monocular-depth residual is automatically reduced for distant markers.

Based on the aforementioned uncertainty model, we construct a normalized Mahalanobis-like distance as the matching cost function. For each pair of candidate matches, the total cost is defined as the sum of the ratios of the geometric residuals to their respective variances:


$$\begin{array}{c} \begin{array}{c} E={\left(\frac{dist(pr,{\text{𝓁}}_{r})}{\sigma_{ep}}\right)}^{\text{2}}+{\left(\frac{{\text{E}}^{\text{sm}}}{\sigma_{\text{mono}}(\text{D})}\right)}^{\text{2}} \end{array} \end{array}$$ (42)

In Eq. (42), the epipolar residual is normalized by the fixed scale σ_ep_, whereas the monocular depth residual is normalized by the distance-dependent uncertainty σ_mono_(D). Therefore, only the monocular uncertainty is updated dynamically for each candidate match.

#### Matching processes.

Candidate correspondences are first generated using the fixed epipolar gating threshold T_ep_ defined above. Candidate correspondences sharing a marker in either image are merged into the same ambiguity group. A group containing only one admissible correspondence is matched directly, whereas a group containing multiple admissible correspondences is processed by exhaustive enumeration.

Consider an ambiguity group containing m candidate markers in the left image and n candidate markers in the right image. The two candidate sets are not required to have equal cardinalities. For each matching hypothesis, all markers in the smaller candidate set are paired one-to-one with an equal-sized subset selected from the larger candidate set. Each marker can participate in at most one correspondence, and only point pairs satisfying the epipolar gating condition are considered. Candidate markers in the larger set that are not included in the current hypothesis are treated as redundant candidates and discarded.

For each candidate pair, the matching cost is calculated using Eq. (42). The total cost of a matching hypothesis H is defined as


$$\begin{array}{c} \begin{array}{c} E_{H}=\sum_{(i,j)\in H}E(P_{l,i},P_{r,j}) \end{array} \end{array}$$ (43)

All feasible one-to-one hypotheses within the ambiguity group are explicitly enumerated, and the optimal hypothesis is selected as


$$\begin{array}{c} \begin{array}{c} H^{*}=\underset{H\in H}{\text{arg}\text{min}}E_{H} \end{array} \end{array}$$ (44)

where ℋ denotes the set of feasible matching hypotheses. The point pairs contained in H^*^ are retained as the final stereo correspondences, whereas the remaining unmatched candidates are discarded.

Let u = min(m,n) and v = max(m,n). In the worst case, the algorithm evaluates v!/(v-u)! hypotheses, and its time complexity is O (uv!/(v-u)!). In the reported ambiguity experiments, at most three candidate markers were involved in each image; therefore, no more than six hypotheses were evaluated for each ambiguity group.

All feasible one-to-one correspondence hypotheses within each ambiguity group are explicitly enumerated, and the hypothesis with the minimum total matching cost is selected.

To intuitively explain the failure mechanism of traditional pure epipolar line matching in long-distance coplanar scenarios, as well as the correction process of the adaptive cost function proposed in this paper, this section extracts a representative frame from a challenging test condition for detailed analysis:

Table 1 demonstrates the error correction mechanism of the proposed algorithm. Traditional greedy epipolar matching relies primarily on local 2D epipolar residuals, which can lead to false matches and large 3D reconstruction errors (up to 161.87 mm). To address this, the proposed method introduces dynamic depth uncertainty to penalize ghost mismatches that yield unreasonable depth estimates. Furthermore, it explicitly enumerates the feasible one-to-one correspondence hypotheses and selects the combination with the minimum global cost, rather than evaluating point pairs independently.As shown, the total cost of the correct matching combination (E_H_ = 1.428) is lower than that of the incorrect one (E_H_ = 97.615).

**Table 1:** Typical cases of the matching algorithm.

Matching Pair	Is Correct	Epipolar Error	Binocular Estimated Position	Monocular Estimation Error	Epipolar Cost	Depth Cost	Total Cost
L1-R1	True	0.191	(−26.09,-0.94,592.51)	16.67	0.228	0.610	0.838
L2-R2	True	0.171	(−65.55,-1.13,586.60)	12.55	0.183	0.407	0.590
L1-R2	False	0.031	(−21.71,-0.76,492.98)	116.29	0.006	29.677	29.683
L2-R1	False	0.194	(−82.14,-1.46,734.99)	161.87	0.236	67.696	67.932

## Experiment

### Experimental system and common evaluation protocol

To validate the effectiveness and real-time performance of the proposed optical tracking algorithm, a high-precision binocular near-infrared visual tracking system was established. The image acquisition module utilizes two industrial-grade CMOS cameras equipped with global shutters. The specific camera parameters and the development platform are detailed in Table 2.

**Table 2:** Hardware parameter list.

Parameter	Specification/ Value
Sensor type	Global shutter
Maximum resolution	1280*1024
Maximum sampling rate	60HZ
Calibrated focal length	1186 pixels
Maximum data transfer rate	1000Mbps
Optical filter	Near-infrared (NIR)/ 850 nm bandpass filter
Computing platform	Intel Core i7-12700H @ 2.3 GHz
Core development framework	C++/ Python/ OpenCV
monocular radius	5.75 mm

The implementation settings for marker detection, ROI tracking, and stereo matching are summarized in Table 3.

**Table 3:** Implementation settings of the proposed method.

Parameter/ definition and unit	Value used
Brightness-mask threshold, Tbright (grayscale intensity)	50
Radial-consistency threshold, Tr (normalized radial-distance RMSE; dimensionless)	0.06
Mean-intensity threshold, TI (grayscale intensity)	170
Candidate contour area (pixels²)	90–1850
Elliptical structuring element, B (width × height; pixels)	3 × 3
Down-sampling factor, n (dimensionless)	2
Global scanning interval, N_scan_ (frames)	10
Maximum inter-frame physical displacement (mm)	30
Epipolar candidate threshold, T_ep_ (pixels)	1

### Center-localization consistency and inter-marker distance accuracy

The contour-fitting analysis should distinguish internal reprojection consistency from independent physical measurement accuracy. The existing 3D-to-2D experiment reconstructs a sphere from the detected image points and reprojects the reconstructed geometry into the same cameras. It therefore evaluates consistency within the calibration and reconstruction pipeline, but it is not an independent validation of absolute 3D accuracy.

To qualitatively and quantitatively verify the accuracy of the contour detection and ellipse fitting algorithms adopted in this paper, a 3D-to-2D Reprojection Verification experiment based on spatial geometric consistency was designed. The specific workflow of this verification mechanism is as follows: First, utilizing the optimal multi-view homologous points filtered by the epipolar matching algorithm, the high-precision 3D position P of the spherical target in the global coordinate system is reconstructed via the optimal triangulation method. Subsequently, with P as the center and the true physical size of the marker as the radius, a virtual ideal sphere model is constructed in 3D space. Finally, by integrating the pre-calibrated camera intrinsic matrix and extrinsic poses, the edge tangent cone of this 3D virtual sphere is reprojected onto the 2D image plane of each camera to calculate the theoretical reprojection ellipse.

Fig 7 presents the reprojection comparison results in the actual image. In the figure, the red contour represents the target’s ellipse boundary directly extracted and fitted during the preprocessing stage, while the green contour represents the theoretical ellipse boundary inversely generated from the 3D sphere. The locally magnified detail in Fig 7B shows that, even in the presence of perspective distortion at the edge of the field of view, the fitted contour and the reprojected contour maintain close geometric agreement. This result supports the stability of the proposed algorithm for sub-pixel edge localization under the tested noisy imaging conditions and indicates consistency across the workflow from 2D contour extraction to 3D spatial reconstruction.

**Fig 7:**
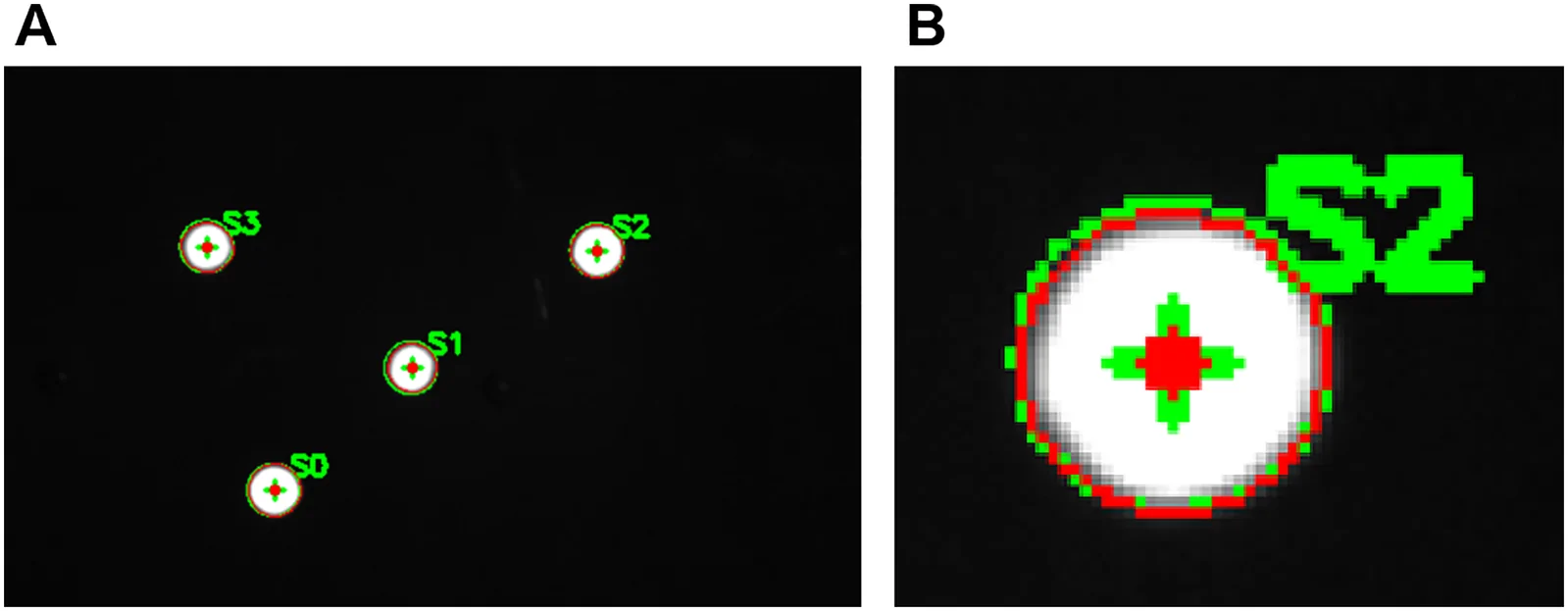
Reprojection of the fitted contours. (A) Global view of the reprojection in the actual image. (B) Locally magnified detail. The fitted target contour and the theoretical contour reprojected from the 3D sphere model are compared.

To further quantitatively evaluate the practical performance of the proposed algorithm from a statistical perspective, we calculated the pixel-level reprojection errors of the reconstructed 3D sphere centers onto the 2D image planes for both the left and right cameras in the binocular vision system, respectively, and compared them with the traditional grayscale centroid extraction method.

As illustrated in Fig 8, subfigures (A) and (B) present the fluctuation curves and mean values of the pixel reprojection errors for the left and right cameras, respectively, across more than 300 independent test samples:

**Fig 8:**
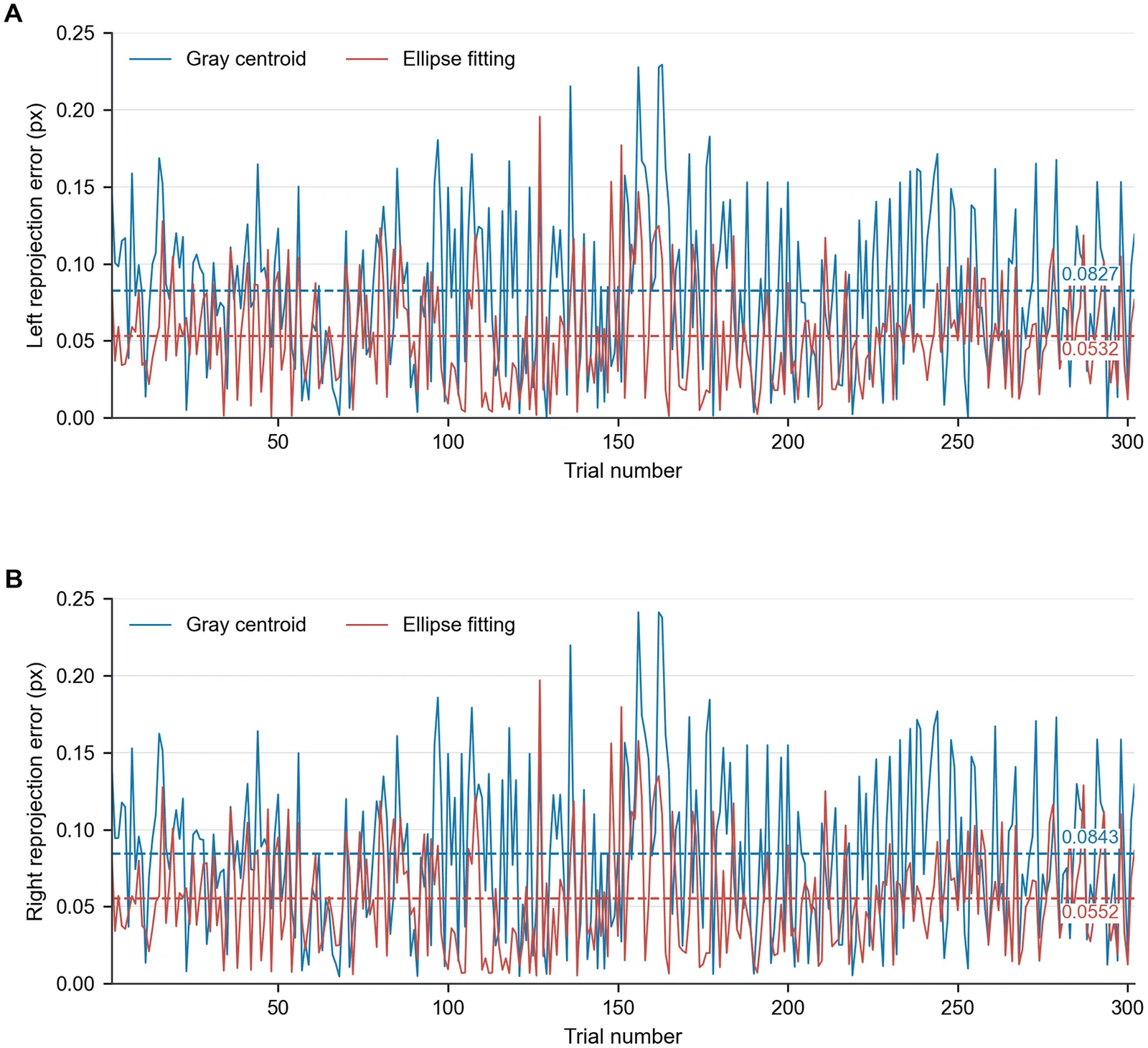
Comparison of reprojection errors between the proposed algorithm and the grayscale-centroid method. (A) Reprojection error of the left camera. (B) Reprojection error of the right camera. Errors were calculated from more than 300 independent test samples.

To further quantitatively evaluate the algorithm’s performance from a statistical perspective, this paper conducts an in-depth analysis of reprojection errors from 300 independent test samples. The results are shown in Table 4. The statistical data indicate that the elliptic fitting algorithm used in this study improves both accuracy and robustness compared with traditional grayscale-centroid methods.

**Table 4:** Statistical values of reprojection error.

Camera	Method	Mean	Std	95% CI	Max
Left camera	Grayscale centroid	0.0827	0.0499	[0.077,0.088]	0.2293
	Proposed method	0.0532	0.0346	[0.049,0.057]	0.1956
Right camera	Grayscale centroid	0.0843	0.0507	[0.079, 0.090]	0.2412
	Proposed method	0.0552	0.035	[0.051, 0.059]	0.197

For the left camera, the reported standard deviation decreased from 0.0499 pixels with the grayscale-centroid method to 0.0346 pixels with the proposed method, a reduction of approximately 30.7%. For the right camera, the reported decrease from 0.0507 to 0.035 pixels was approximately 30.9%. The maximum reprojection error remained below 0.2 pixels for the proposed method in the reported samples.

To evaluate the influence of marker-center localization on the final three-dimensional measurement accuracy, the proposed four-parameter constrained ellipse-fitting method was compared with the unconstrained ellipse-fitting method implemented by OpenCV (cv2.fitEllipse) and the grayscale-centroid method. The same 150 stereo observations were processed using identical calibration parameters, contour inputs, and correspondence labels so that the comparison isolated the effect of center localization.

For each method, the three-dimensional coordinates of the two spherical markers were reconstructed, and their Euclidean separation was compared with the independently measured reference distance. The absolute distance error for method m in observation k was defined as


$$\begin{array}{c} \begin{array}{c} e_{k}^{\left(m\right)}=|{\hat{L}}_{k}^{\left(m\right)}-L_{ref}| \end{array} \end{array}$$ (45)

where L̂_k_^(m)^ is the reconstructed inter-marker distance for method m in observation k, and L_ref_ is the externally measured reference distance.

Fig 9 shows the distributions of absolute inter-marker distance errors for the three center-localization methods.

**Fig 9:**
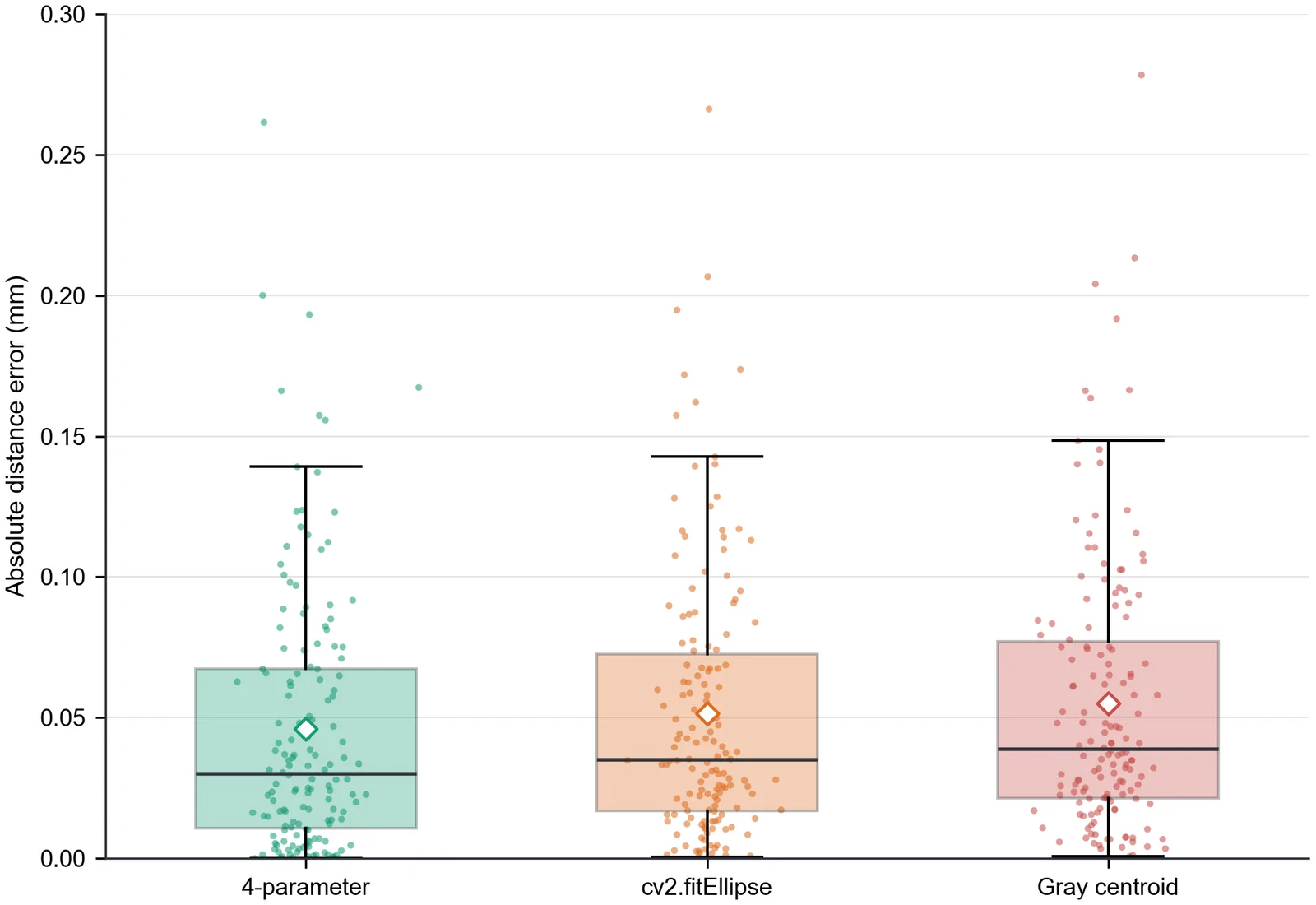
Distribution of absolute inter-marker distance errors obtained using three center-localization methods. Each point represents one measurement (N = 150 per method); the boxes show the interquartile ranges, the horizontal lines indicate the medians, and the diamonds indicate the mean errors.

Table 5 summarizes the corresponding absolute inter-marker distance error statistics. The proposed four-parameter constrained fit achieved the lowest MAE of 0.0458 mm and the lowest RMSE of 0.0659 mm. The corresponding values were 0.0515 and 0.0701 mm for OpenCV fitEllipse and 0.0551 and 0.0732 mm for the grayscale-centroid method.

**Table 5:** Absolute inter-marker distance errors obtained using three center-localization methods (all values in mm; N = 150 per method).

Method	MAE	RMSE	SD	95% CI of MAE	Max error
Four-parameter constrained fit	0.0458	0.0659	0.0474	[0.0382, 0.0535]	0.2618
OpenCV fitEllipse	0.0515	0.0701	0.0477	[0.0438, 0.0592]	0.2664
Grayscale centroid	0.0551	0.0732	0.0483	[0.0473, 0.0629]	0.2786

Relative to OpenCV fitEllipse, the proposed method reduced the MAE and RMSE by approximately 10.9% and 6.0%, respectively. Compared with the grayscale-centroid method, the corresponding reductions were approximately 16.8% and 10.0%. The maximum absolute error was also reduced from 0.2664 mm and 0.2786 mm to 0.2618 mm. However, the SD values were similar for all three methods, ranging from 0.0474 to 0.0483 mm. Thus, the main improvement was a reduction in average distance error, while the overall dispersion remained comparable. These descriptive results indicate that the geometric constraint in the four-parameter model improved inter-marker distance measurement accuracy under the tested conditions.

### Distance-dependent validation of monocular uncertainty

To evaluate the predicted increase in monocular depth uncertainty with working distance, markers were acquired at six depth planes from 500 to 1100 mm. Stereo reconstruction was used as a reference measurement.The system calculated the 2D epipolar residual and the monocular depth deviation for every accepted match.

Fig 10 intuitively quantifies the statistical distribution of the errors in the form of box plots. Fig 10A shows that the epipolar residuals remained stable throughout the tested workspace, with the median maintained at approximately 0.1 px and the maximum residual below 0.2 px. This result supports the robustness of the sub-pixel extraction procedure and indicates that 2D feature-extraction errors contributed only limited interference to the 3D calculation under these conditions. In stark contrast, the monocular depth deviations in Fig 10B present a prominent nonlinear divergent characteristic: as the reference distance increases, the height of the error box (interquartile range) and the span of the whiskers stretch drastically, with the maximum residual at long distances exceeding 35 mm.

**Fig 10:**
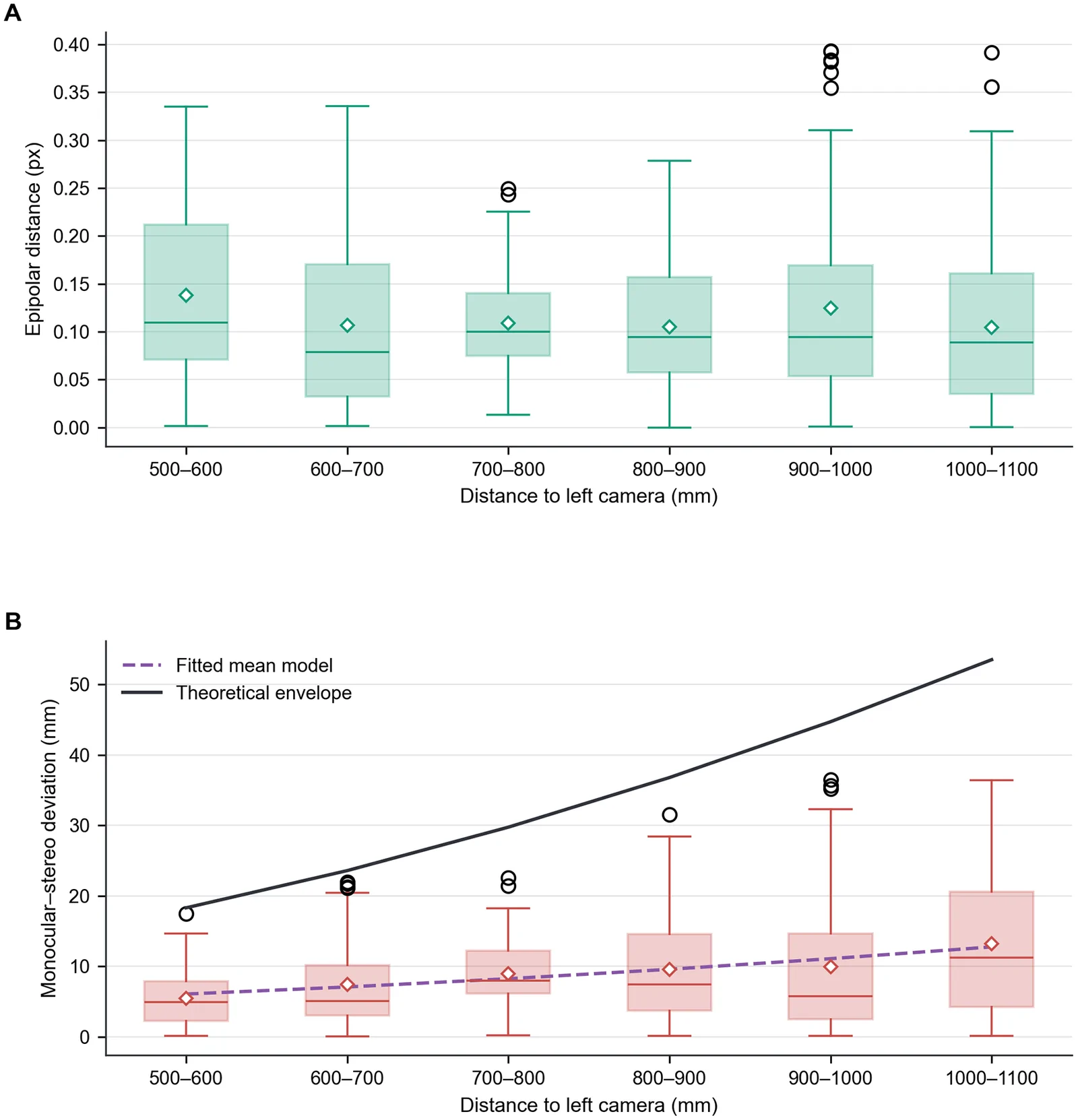
Statistical distribution and theoretical validation of measurement errors as a function of reference distance. (A) Box plots of 2D epipolar residuals. (B) Box plots of monocular depth deviations with the fitted mean curve and theoretical uncertainty envelope.

To establish the connection between the experimental results and the theoretical analysis, Fig 10B superimposes the theoretical uncertainty model given in Eq. (41). The purple dashed line in the figure represents the fitted curve of the mean values for each interval, corresponding to an effective pixel error of δp = 0.058px, which highly coincides with the system’s true sub-pixel extraction accuracy. Meanwhile, the dark blue solid line is the theoretical envelope plotted by substituting the maximum extraction error (δp = 0.3px), which closely bounds the maximum values and outliers across the tested depths.

The approximately constant epipolar residual and the increasing monocular depth deviation are consistent with the distance-dependent uncertainty model over the tested range. This result supports the selected normalization strategy within the experimental workspace; it does not establish physical inevitability or independent absolute localization accuracy.

### Stereo-matching comparison under ghost-marker ambiguity

As illustrated in Fig 11, a custom-made asymmetric cross-shaped surgical instrument carrying five spherical markers was used in this experiment. Three collinear markers were arranged along each arm of the cross, with the central marker shared by the two arms. The two arms had different lengths, resulting in an asymmetric marker configuration. The ideal coordinates of the five markers were predefined in the tool coordinate system according to the structural dimensions of the instrument.

**Fig 11:**
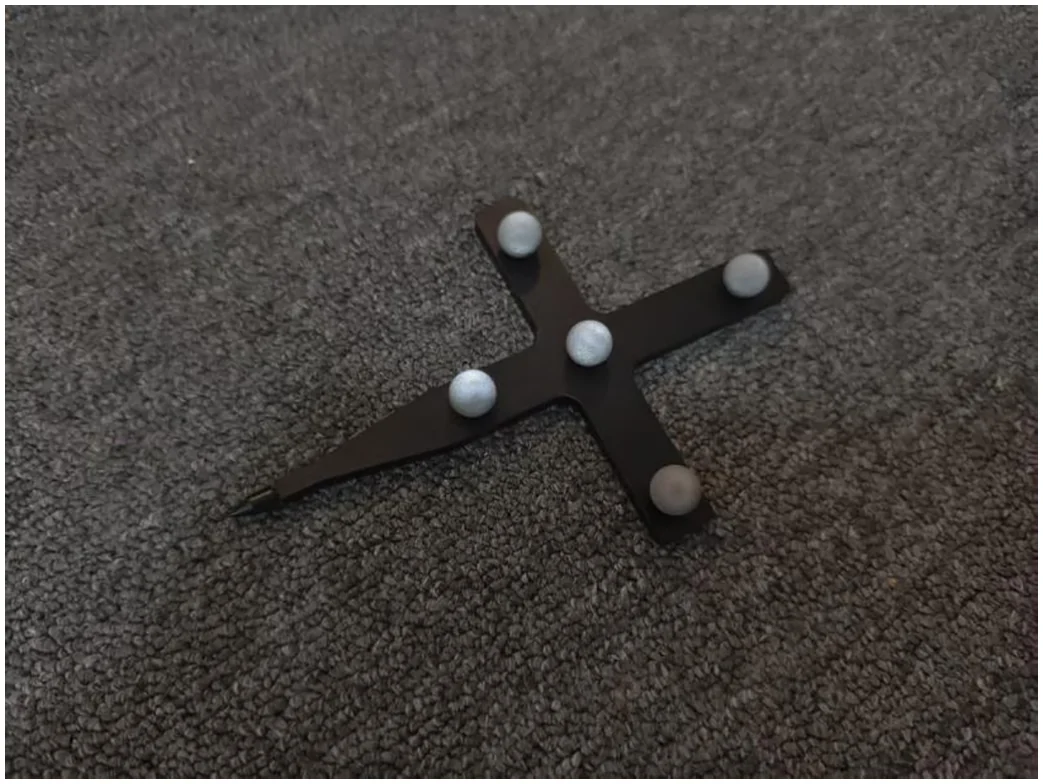
Custom-designed surgical instrument. The probe contains five spherical markers arranged asymmetrically and serves as the rigid-body topology prior for evaluating matching robustness.

To generate controlled ghost-marker ambiguity, the instrument was positioned such that one arm containing three collinear markers was approximately parallel to the stereo-camera baseline, defined as the line connecting the optical centers of the left and right cameras. The marker pairs along this arm were defined as baseline-parallel marker pairs, whereas those along the other arm were defined as baseline-perpendicular marker pairs. Because the two arms shared the central marker, both types of marker pairs were present simultaneously in every acquired frame. The instrument was subjected to small-amplitude oscillatory motion while maintaining this approximate orientation, causing the projections of the baseline-parallel markers to lie on or near the same epipolar line and thereby deliberately generating stereo-correspondence ambiguity.

The same image sequences were subsequently analyzed at two complementary levels. First, the internal discriminative ability of the proposed matching cost was evaluated by comparing all feasible correspondence hypotheses for the three collinear markers. Second, the end-to-end reconstruction performance of three stereo-matching methods was evaluated by comparing their reconstructed marker coordinates with the ideal coordinates of the instrument after least-squares rigid registration.

The first analysis examined whether the proposed cost function could distinguish the correct correspondence from the incorrect correspondence hypotheses generated by epipolar overlap. In each selected frame, the three collinear markers on the baseline-parallel arm formed an ambiguity group because their projections lay on or close to the same epipolar line. For three markers in the left and right images, a total of 3! = 6 one-to-one correspondence hypotheses were possible. All six hypotheses were explicitly enumerated, and the total matching cost of each hypothesis was calculated using the proposed epipolar-plus-monocular-depth cost defined in Eq. (42).

The correct correspondence hypothesis was identified according to the known marker identities and reference geometry of the cross-shaped instrument and was denoted Case 6. The remaining five incorrect hypotheses were denoted Cases 1–5. Fig 12 shows the matching costs of the six correspondence hypotheses and illustrates the internal discriminative ability of the proposed cost function.

**Fig 12:**
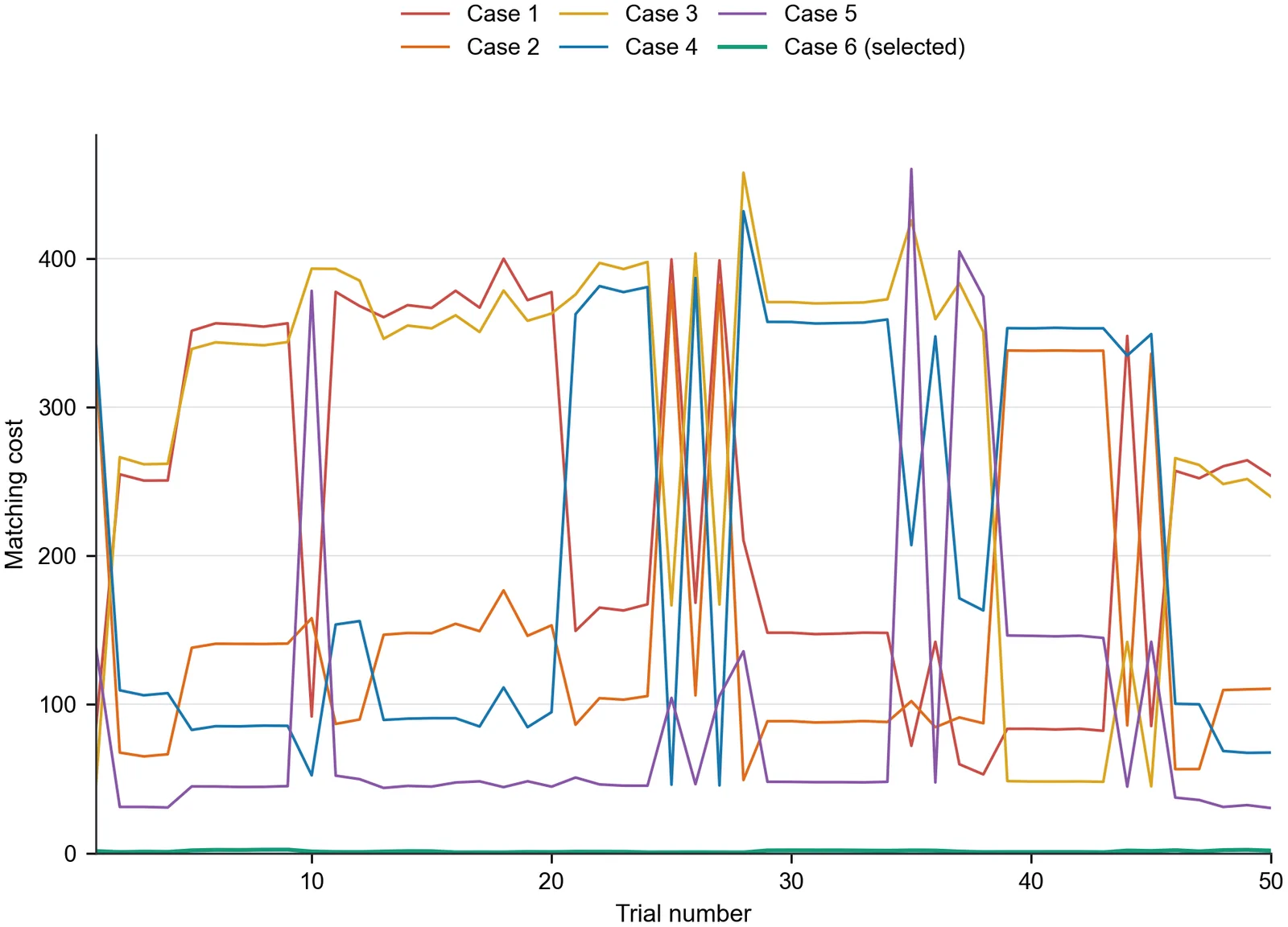
Function values of matching cost under different pairing cases. Case 6 denotes the correct matching configuration, whereas Cases 1-5 denote incorrect configurations; lower cost indicates a more plausible matching result.

Across 50 independent acquisition trials, each trial produced six related matching-cost values, one for each correspondence hypothesis. As shown in Fig 12, the correct hypothesis, Case 6, consistently produced the lowest matching cost. Its mean cost was 1.1591, compared with mean values ranging from 90.8335 to 295.7596 for the incorrect hypotheses. This separation indicates that the monocular-depth term substantially penalized the geometrically implausible correspondences that could not be distinguished reliably using the epipolar residual alone.

The results show that the correct matching scheme (Case 6) had the lowest cost among the six tested cases. The mean matching cost of Case 6 was 1.1591, which was substantially lower than those of the incorrect matching cases (Cases 1–5).

In terms of dispersion, the standard deviation (SD = 0.5905) and maximum value of Case 6 were both low relative to the incorrect cases, indicating that the objective function was more consistent for the correct matching parameters. In contrast, the incorrect matching cases showed larger error fluctuations, with maximum errors far exceeding the mean value of the correct match. This statistical separation supports the effectiveness of the proposed cost function for reducing ghost-point interference in the tested coplanar-ambiguity scenarios.

Table 6 summarizes the matching-cost statistics for the six correspondence hypotheses. This analysis compares correspondence hypotheses rather than different stereo-matching algorithms.

**Table 6:** Statistical values of the matching cost for different correspondence hypotheses.

Case	Mean	SD	Max	Min
1	232.4693	118.4984	400.1337	52.9441
2	151.2968	97.7543	382.9447	49.1691
3	295.7596	120.5867	458.0633	44.2631
4	209.9573	134.7913	432.1359	45.5320
5	90.8335	101.3666	460.5334	30.3058
6	1.1591	0.5905	2.2750	0.4177

The second analysis evaluated whether the separation observed in the matching-cost space translated into improved three-dimensional reconstruction accuracy. The same image sequences and corrected marker centers used in the preceding cost-function analysis were supplied to three stereo-matching methods: conventional epipolar-only matching, MSRC [19], and the proposed epipolar-plus-monocular-depth method. Thus, differences in the reconstruction results were attributable to the stereo-matching strategies rather than to marker detection or center localization.

For each frame, the five reconstructed markers were aligned with their corresponding ideal positions in the tool coordinate system using least-squares rigid registration. The Euclidean distance between each registered marker and its corresponding ideal position was then calculated as the point-registration error.

Because the baseline-parallel and baseline-perpendicular marker groups were simultaneously present on the cross-shaped instrument, their registration errors were evaluated separately from the same acquisition sequences. Fig 13(A) presents the errors associated with the baseline-perpendicular marker group, whereas Fig 13(B) presents those associated with the baseline-parallel marker group. The latter represents the more challenging condition because the projections of the three collinear markers lie on or near the same epipolar line.

**Fig 13:**
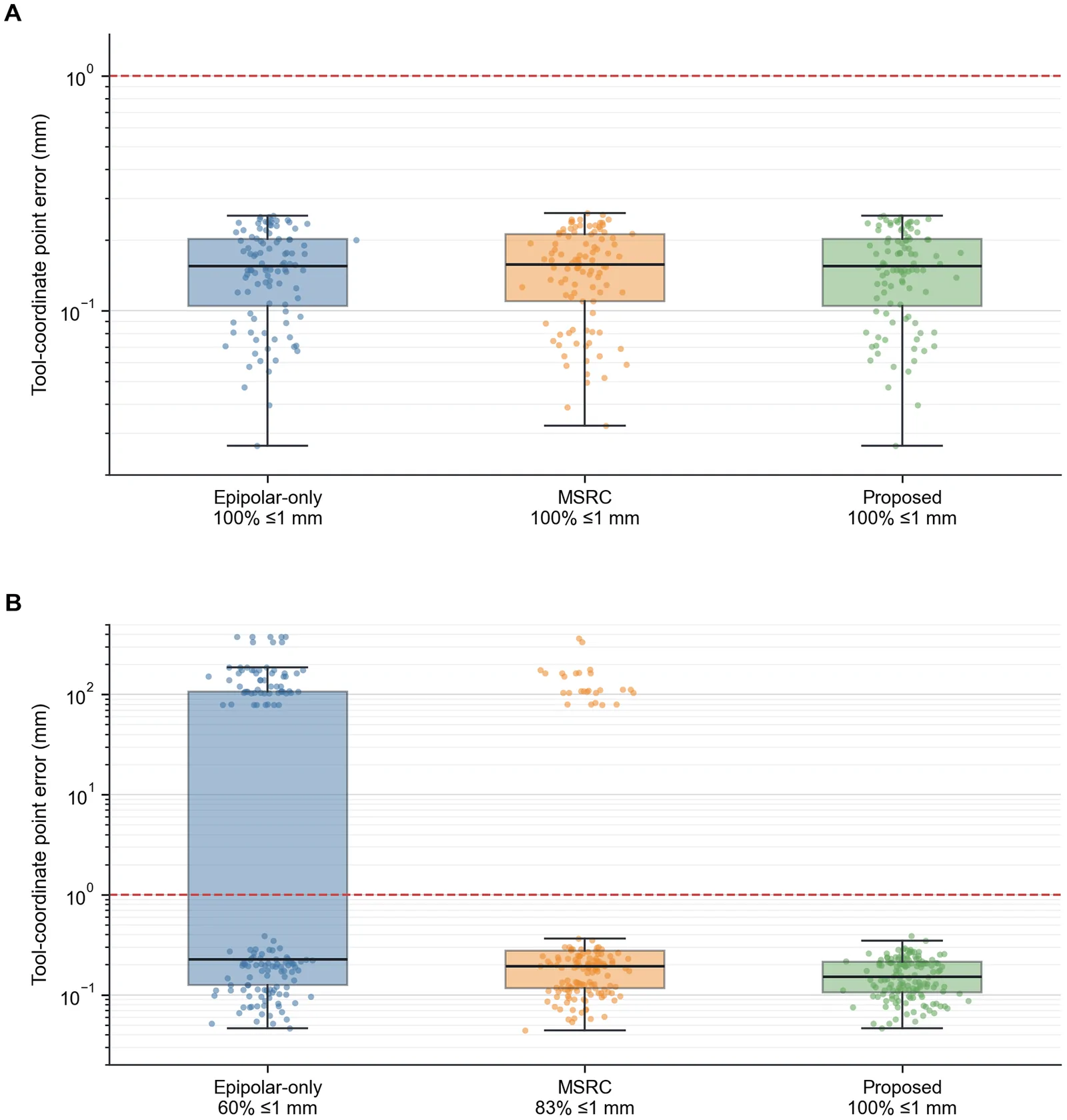
Comparison of point-registration errors under ghost-marker ambiguity using epipolar-only matching, MSRC, and the proposed method. (A) Registration errors for the baseline-perpendicular marker group. (B) Registration errors for the baseline-parallel marker group, for which the three collinear marker projections lie on or near the same epipolar line. The results in both panels were obtained from the same acquisition sequences. The reconstructed marker sets were aligned with the ideal tool geometry using least-squares rigid registration before the point-wise Euclidean errors were calculated. The boxes indicate the interquartile range, the center lines indicate the medians, and the overlaid points represent individual observations. The red dashed line denotes the 1 mm error threshold, and the percentages indicate the proportion of observations with errors not exceeding 1 mm. Both vertical axes are shown on a logarithmic scale.

Fig 13 compares point-registration errors between fitted markers and reconstructed markers using the traditional epipolar-constraint method and the proposed algorithm. In the dataset containing only vertical ghost markers (Fig 13A), the two curves almost overlap, indicating that both methods achieved similar registration accuracy under this condition, with a mean error of 0.246 mm. When horizontal ghost markers were introduced (Fig 13B), the traditional epipolar-constraint method was affected by epipolar overlap and showed large registration-error fluctuations, with repeated error peaks exceeding 300 mm and the maximum error approaching 380 mm. In contrast, the proposed algorithm maintained a low sub-millimeter error level, with a mean error of 0.233 mm. These results indicate that the proposed method improves robustness under horizontal ghost-marker interference, where epipolar-only matching is more likely to fail.

To summarize the accuracy and computational efficiency of the three stereo-matching methods, Fig 14 presents their frame-level matching accuracy and execution-time distributions over the same 50 stereo frames. A frame was considered correctly matched only when all five markers were successfully reconstructed and the registration error of every marker did not exceed 1 mm after least-squares rigid registration.

**Fig 14:**
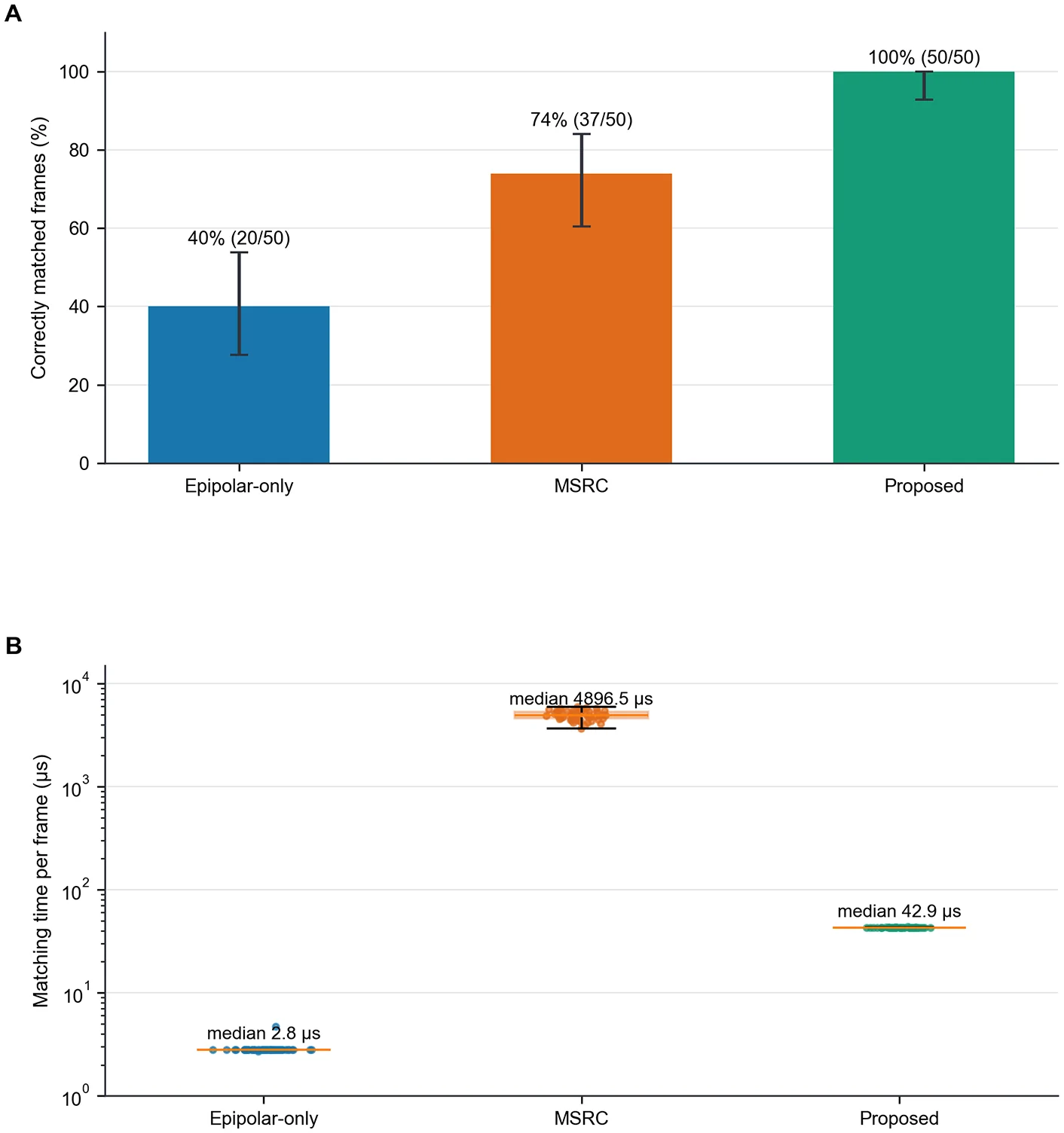
Frame-level matching accuracy and computational cost of the three stereo-matching methods over the same 50 stereo frames. (A) Percentage and number of correctly matched frames. A frame was considered correct only when all five markers were successfully reconstructed and the registration error of every marker did not exceed 1 mm after least-squares rigid registration. Error bars indicate the Wilson 95% confidence intervals. (B) Per-frame execution-time distributions of the stereo-matching stage. Each point represents one frame, the center lines indicate the medians, and the boxes indicate the interquartile ranges. The vertical axis in (B) is displayed on a logarithmic scale.

As shown in Fig 14A, epipolar-only matching correctly processed 20 of the 50 frames, corresponding to an accuracy of 40.0%, whereas MSRC correctly processed 37 frames and achieved an accuracy of 74.0%. The proposed method correctly processed all 50 frames and achieved an accuracy of 100.0%. Fig 14B shows that the median matching times of epipolar-only matching, the proposed method, and MSRC were 2.80 μs, 42.9 μs, and 4.90 ms per frame, respectively. Although the proposed method required more computation than epipolar-only matching, its median execution time remained below 0.05 ms and was approximately 114 times lower than that of MSRC. The proposed method therefore achieved the best accuracy-efficiency balance under the tested ghost-marker ambiguity condition.

### Parameter sensitivity analysis

Three empirically specified thresholds directly affect the contour-based marker-processing pipeline. The brightness-mask threshold T_bright_ determines the region within which edge evolution and contour filling are performed and may therefore affect the fitted ellipse dimensions and the resulting monocular distance estimate. In contrast, the radial-consistency threshold T_r_ and the mean-intensity threshold T_I_ determine whether an extracted contour is retained as a marker candidate and primarily affect false-positive and false-negative detections. We therefore evaluated T_bright_ using monocular distance error and evaluated T_r_ and T_I_ using detection precision, recall, and F1-score. During each experiment, only the parameter under investigation was varied, while all other parameters were held fixed.

To evaluate the sensitivity of monocular distance estimation to T_bright_, the threshold was varied from 40 to 60 in increments of 5. The same observations were reprocessed at every threshold value, while the remaining contour-extraction and ellipse-fitting parameters were held fixed. The Euclidean camera-to-marker distance obtained by stereo triangulation was used as the reference. Because monocular estimation uncertainty increases with distance, the observations were divided into near (0.50–0.70 m), medium (0.70–0.90 m), and far (0.90–1.10 m) groups. For each threshold and distance group, the mean absolute error (MAE) between the monocular estimate and the triangulated reference was calculated. Error bars represent one standard deviation of the absolute errors.

As shown in Fig 15, the monocular distance error exhibited a U-shaped dependence on T_bright_ in all three distance groups. The minimum MAEs were obtained at T_bright_ = 50: 6.70 mm for the near group, 9.65 mm for the medium group, and 12.57 mm for the far group. Lower thresholds of 40–45 tended to underestimate the marker distance, whereas thresholds of 55–60 produced increasing positive bias, particularly in the far group. Accordingly, T_bright_ = 50was selected for the subsequent experiments.

**Fig 15:**
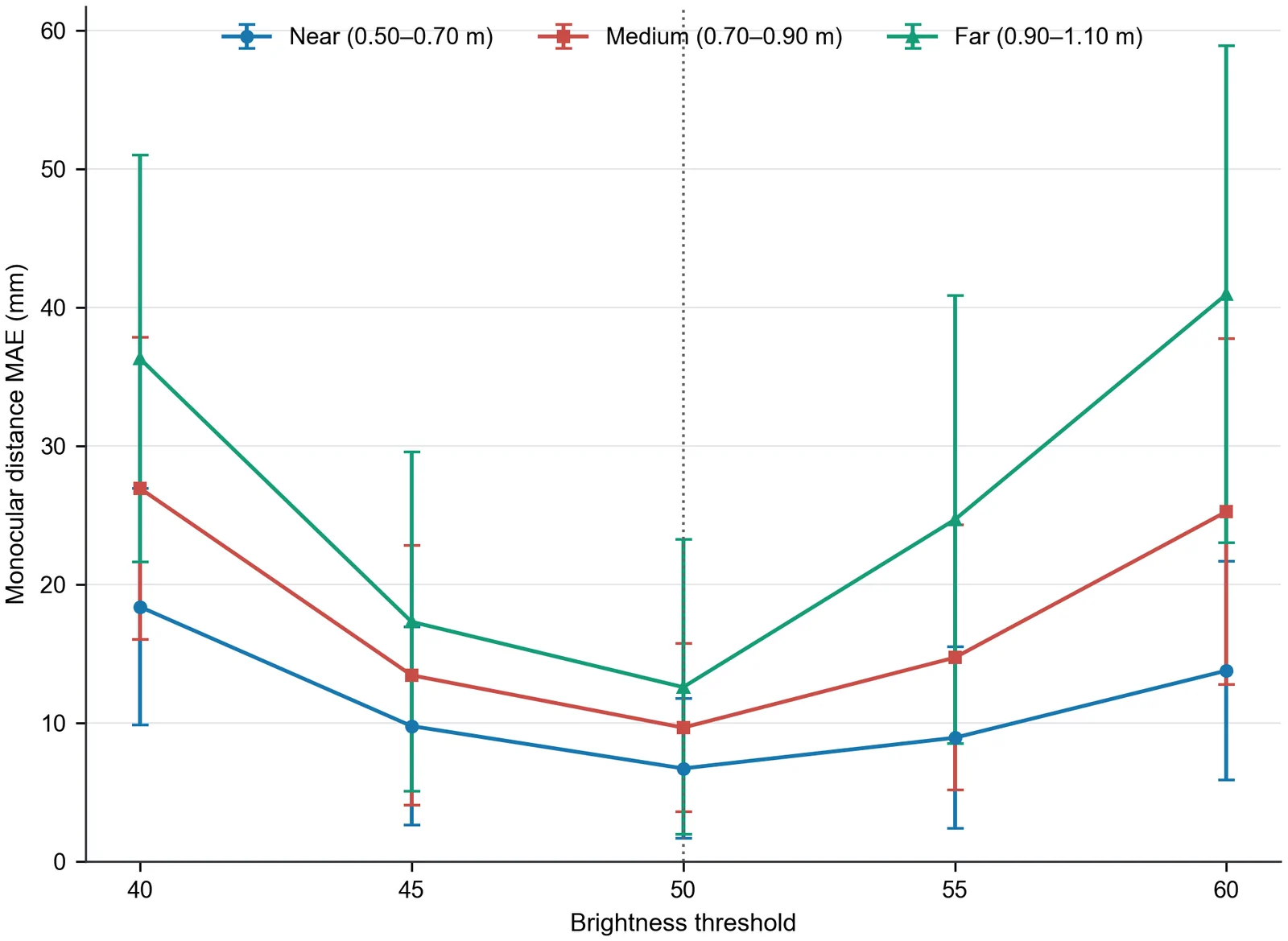
Sensitivity of monocular distance estimation to the brightness-mask threshold T_bright_. The monocular distance MAE is reported for the near (0.50–0.70 m), medium (0.70–0.90 m), and far (0.90–1.10 m) distance groups. The triangulated Euclidean camera-to-marker distance was used as the reference. Error bars indicate one standard deviation of the absolute errors. The vertical dotted line denotes the selected value (T_bright_ = 50).

To evaluate the radial-consistency threshold T_r_ and the mean-intensity threshold T_I_, a controlled reflective-interference scene was constructed. Multiple metallic objects and near-infrared reflective patches were deliberately placed around the spherical marker tool. Fig 16A shows their physical arrangement in a color photograph. Fig 16B shows the scene captured by the experimental camera equipped with a near-infrared bandpass filter; the colored markers in this panel are overlays of the algorithm’s detection results. Under near-infrared illumination, the interfering objects produced bright regions that could be confused with marker candidates. The images therefore illustrate the conditions under which the two candidate-screening thresholds were evaluated.

**Fig 16:**
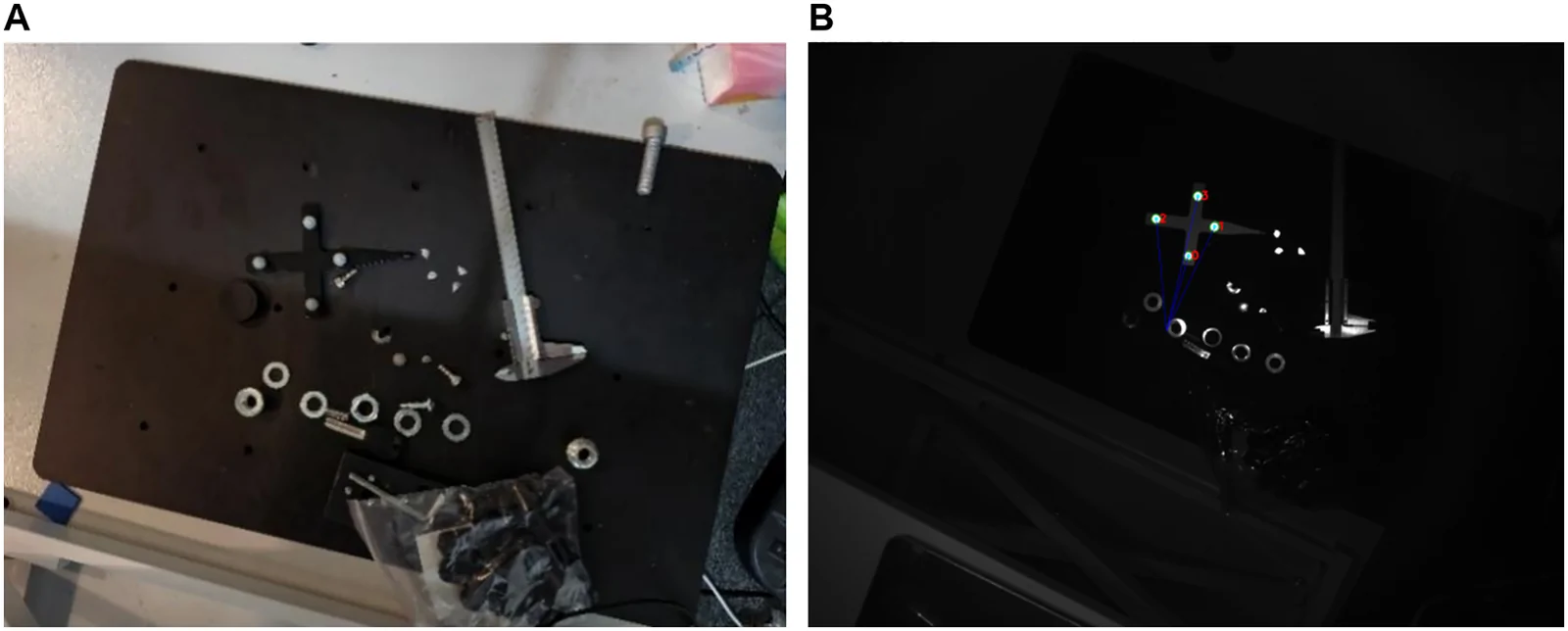
Controlled reflective-interference environment used for the parameter-sensitivity experiments. (A) Color photograph showing the physical arrangement of the marker tool, metallic distractors, and near-infrared reflective patches. The color image was acquired only to document the experimental setup and was not used by the detection algorithm. (B) Corresponding image acquired by the experimental tracking camera equipped with a near-infrared bandpass filter. Bright spots, annular reflections, and irregular high-intensity regions were produced by the deliberately introduced distractors. The colored overlays indicate the marker centers correctly identified by the proposed algorithm.

The same 128 near-infrared images were used to evaluate both candidate-screening thresholds. Four spherical markers in each image were manually annotated, and detected centers were matched one-to-one with the annotated centers using a maximum distance tolerance of **3 pixels**. Matched detections were counted as true positives (TP), unmatched detections as false positives (FP), and unmatched annotations as false negatives (FN). For each threshold setting, TP, FP, and FN were accumulated across the images to calculate precision, recall, and F1-score.

A one-parameter-at-a-time procedure was used. To evaluate the radial-consistency threshold T_r_, its value was varied from 0.04 to 0.08 in increments of 0.01, while T_bright_ = 50, T_I_ = 170, and the other processing parameters were held fixed. To evaluate T_I_, its value was varied from 150 to 190 in increments of 10, while T_bright_ = 50, T_r_ = 0.06, and the other parameters were held fixed. Each setting was applied to the same images, so the resulting changes in detection performance could be attributed to the threshold being varied. The results are shown in Fig 17A and Fig 17B, respectively.

**Fig 17:**
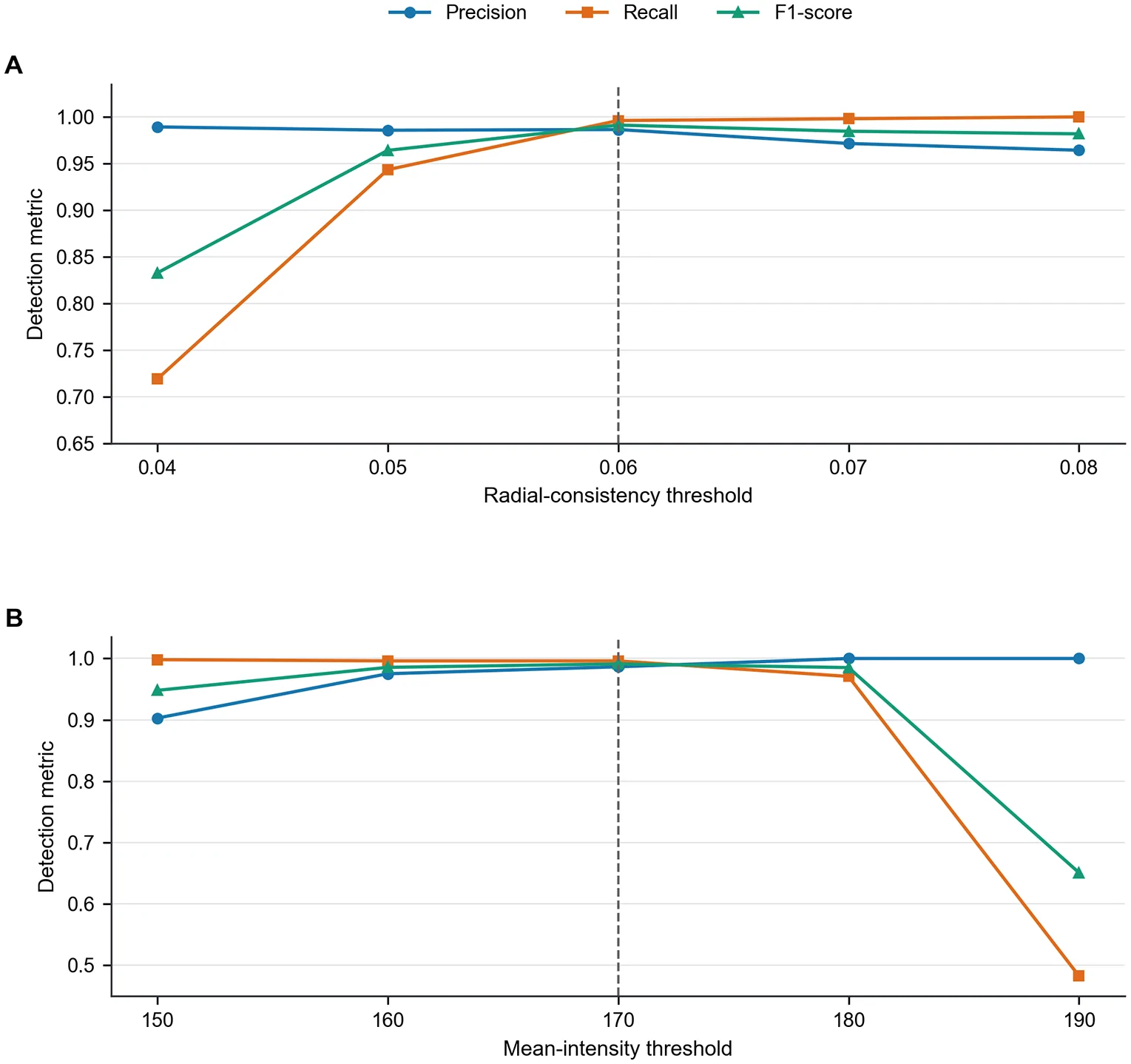
Sensitivity of marker detection to the candidate-screening thresholds. Precision, recall, and F1-score are shown as functions of (A) the radial-consistency threshold T_r_ and (B) the mean-intensity threshold T_I_. During each experiment, only the parameter under investigation was varied, while all other parameters were held fixed. The vertical dashed lines denote the selected values T_r_ = 0.06 and T_I_ = 170.

As shown in Fig 17A, an excessively strict radial-consistency threshold rejected valid marker contours and reduced recall, whereas relaxing the threshold increased recall but gradually introduced additional false-positive detections. The maximum F1-score of 0.9913 was obtained at T_r_= 0.06, which was therefore selected for the subsequent experiments.

As shown in Fig 17B, increasing T_I_ initially reduced false-positive detections while maintaining a high recall. The highest F1-score of 0.9913 was obtained at T_I_= 170. A further increase to 190 substantially reduced recall because valid markers with relatively low mean intensity were rejected. Therefore, T_I_= 170 was selected.

### Marker detection under illumination interference

To investigate the influence of time-varying illumination in a challenging reflective environment, 50 paired experimental groups were acquired. Multiple metallic surgical instruments were placed around the target to introduce interference from irrelevant objects and reflective surfaces. Within each group, the camera, target, and surgical-tool configurations were kept unchanged. A baseline image and an image under additional white-LED illumination were acquired consecutively by alternately switching the LED source off and on. Thus, each group contained a pair of measurements acquired under identical geometric configurations but different illumination conditions, resulting in 100 images in total.

The experiment evaluated marker detection, false detection, and three-dimensional reconstruction consistency. For each image, the number of detected marker spheres, missed marker spheres, and false-positive detections was recorded. Because accurate ground-truth three-dimensional coordinates were unavailable, the experiment did not evaluate absolute reconstruction accuracy. Instead, two consistency metrics were employed: (1) the rigid-fit residual RMSE of the reconstructed tool coordinates and (2) the three-dimensional discrepancy between the monocular and stereo reconstruction results. A reconstruction was considered valid when its rigid-fit residual RMSE was below the predefined threshold 1 mm. For both metrics, the results obtained under baseline and white-LED illumination were compared within each paired group.

All expected marker spheres were successfully detected in every acquired image under both baseline and white-LED illumination. No missed detections or false-positive detections were observed. Therefore, the frame-level detection success rate was 100%, and the false-positive frame rate was 0% under both illumination conditions. These results also demonstrate that the system successfully rejected interference from the surrounding metallic instruments and reflective surfaces in the tested scenes.

As shown in Fig 18A, the mean rigid-fit residual RMSE was 0.158 mm under baseline illumination and 0.155 mm under white-LED illumination, with maximum values of 0.288 and 0.330 mm, respectively. All residuals remained within the predefined valid range. Fig 18B shows that the mean monocular–stereo 3-D discrepancies were 10.649 and 10.832 mm, while the corresponding maximum values were 26.295 and 26.047 mm. The closely overlapping curves indicate that the additional white-LED illumination did not produce an appreciable degradation in marker detection or reconstruction consistency under the tested multi-instrument reflective environment.

**Fig 18:**
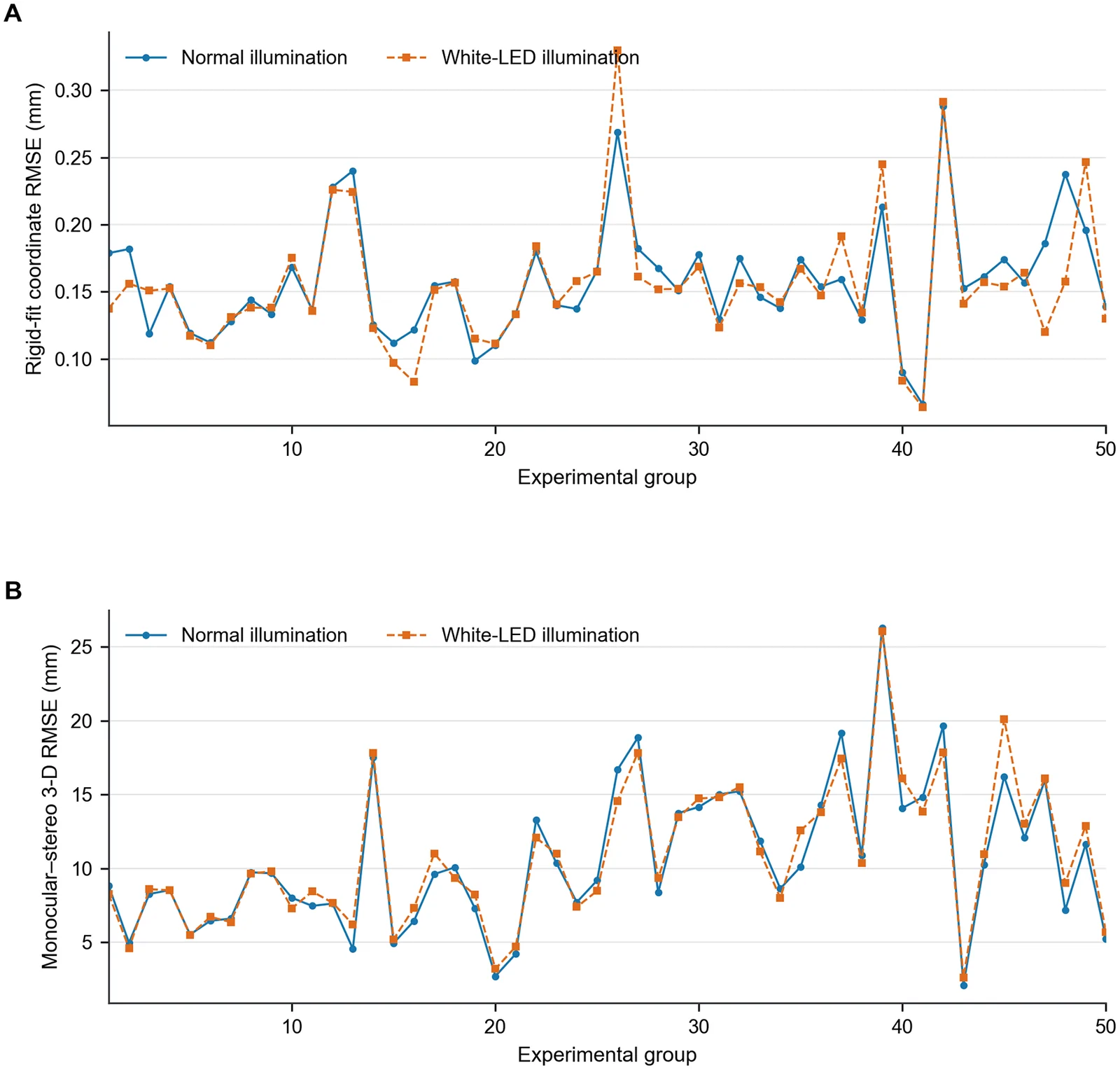
Reconstruction consistency under baseline and alternating white-LED illumination in a multi-instrument reflective environment. (A) Rigid-fit residual RMSE of the reconstructed tool coordinates. (B) Three-dimensional discrepancy between the monocular and stereo reconstruction results. Each experimental group contains two consecutive measurements acquired with an unchanged geometric configuration.

### Algorithmic processing latency and ROI acceleration

The computational efficiency of the stereo-matching algorithms was evaluated separately in Fig 14 using the same stereo frames and computing platform. The present experiment focuses on the acceleration achieved by image downsampling and adaptive region-of-interest processing. Accordingly, the three configurations compared in this subsection are ablations of the same processing pipeline rather than different stereo-matching algorithms. They use the same marker-extraction, stereo-matching, and three-dimensional reconstruction procedures and differ only in the spatial image-processing strategy.

All runtime experiments were performed on the computing platform listed in Table 2. The C++ implementation was built in x64 Release mode using Microsoft Visual C++ (MSVC) 19.29.30159 (Visual Studio 2019, v142 toolset) and OpenCV 4.5.5. No application-level CPU multithreading or GPU acceleration was used.The same synchronized stereo sequences were processed by all three configurations. The timing interval started after the left and right images were available in memory and ended after the three-dimensional marker coordinates were obtained. Camera exposure, image transfer, visualization, file input/output, and data logging were excluded from the reported processing time. For each marker configuration, 50 warm-up frames were discarded, and processing times from the subsequent 80 stereo frame pairs were analyzed. The mean, standard deviation, median and interquartile range were calculated separately for each condition.

Fig 19 compares the time required to obtain the three-dimensional marker coordinates as the number of markers increased from 1 to 15. Fig 19A presents the median processing time and interquartile range, whereas Fig 19B presents the mean and standard deviation. Across all tested marker counts, adaptive ROI processing had the lowest latency, followed by downsampled global search and full-resolution global search. With one marker, the mean processing times were 28.86, 16.32, and 2.20 ms per stereo frame pair, respectively. With 15 markers, they increased to 39.58, 22.31, and 16.52 ms, respectively. At 15 markers, adaptive ROI processing reduced the mean latency by 58.3% relative to full-resolution global search and by 26.0% relative to downsampled global search. The median results showed the same ordering.

**Fig 19:**
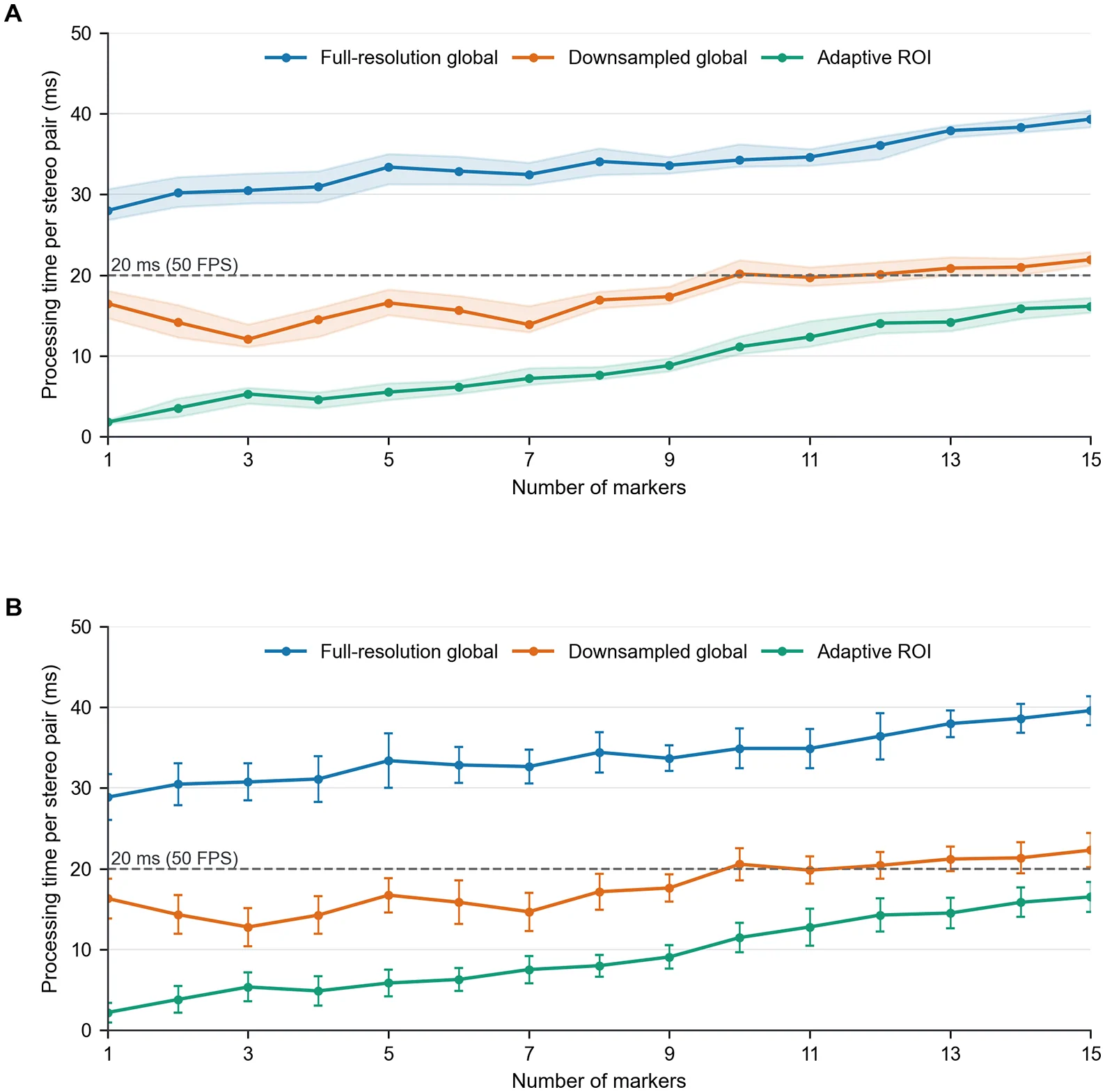
Processing time of three-dimensional reconstruction under different marker counts. (A) Median processing time; shaded regions indicate the interquartile range. (B) Mean processing time; error bars indicate one standard deviation. Each point represents 80 stereo frame pairs. The adaptive ROI samples include periodic coarse-search frames. The dashed line indicates 20 ms per stereo frame pair, equivalent to an algorithmic processing throughput of 50 frame pairs per second.

Across the tested range of 1–15 markers, the mean algorithmic processing time with adaptive ROI remained below 20 ms per stereo frame pair. At 15 markers, the mean was 16.52 ms, whereas the 95th-percentile latency was 21.20 ms; therefore, individual frames did not always meet a 20-ms processing deadline. These measurements start after the stereo images are available in memory and exclude camera exposure and image transfer. They support the computational benefit of adaptive ROI processing, but should not be interpreted as a guaranteed end-to-end system frame rate.

## Conclusion

Marker-based optical tracking requires accurate marker localization and unambiguous stereo correspondence, particularly when reflective interference or specific marker arrangements create false matching candidates. This study developed a tracking strategy that combines contour-based marker extraction, contour-derived monocular depth priors, uncertainty-normalized stereo matching, and adaptive region-of-interest (ROI) processing. The main findings are summarized as follows:

The contour-based method improved marker-center localization and inter-marker distance measurement under the tested conditions. Boundary evolution and circularity filtering were combined with four-parameter constrained ellipse fitting to localize spherical markers. Compared with the grayscale-centroid method, the proposed method reduced the mean reprojection errors from 0.0827 to 0.0532 pixels in the left camera and from 0.0843 to 0.0552 pixels in the right camera. These reprojection results demonstrate improved consistency within the calibrated imaging and reconstruction pipeline. In a separate experiment involving 150 stereo observations and an independently measured inter-marker reference distance, the proposed method achieved a mean absolute distance error of 0.0458 mm, compared with 0.0515 mm for OpenCV fitEllipse and 0.0551 mm for the grayscale-centroid method. In the paired illumination experiment, all expected markers were detected without missed or false detections in the 100 images acquired under baseline and additional white-LED illumination.

The monocular-depth prior helped resolve ghost-marker ambiguity that could not be reliably addressed by epipolar information alone. The method estimates monocular depth from the perspective deformation of spherical-marker contours and combines this information with epipolar residuals in an uncertainty-normalized matching cost. For the six feasible correspondence hypotheses evaluated in each of 50 ambiguity trials, the correct hypothesis consistently received the lowest cost. In the frame-level comparison using the same stereo image sequences, the proposed method met the predefined correctness criterion in all 50 frames, whereas epipolar-only matching and MSRC met it in 20 and 37 frames, respectively. These results support the effectiveness of the depth-informed matching strategy for the tested baseline-parallel marker configuration.

Adaptive ROI processing reduced the computational cost of the complete marker-processing pipeline. Across the tested range of 1–15 markers, its mean algorithmic processing time remained below 20 ms per stereo frame pair. With 15 markers, the mean time was 16.52 ms, representing reductions of 58.3% relative to full-resolution global search and 26.0% relative to downsampled global search. However, the 95th-percentile time at 15 markers was 21.20 ms, so the method did not meet a 20-ms deadline in every frame. Moreover, these measurements excluded camera exposure and image transfer and should not be interpreted as a guaranteed end-to-end system frame rate.

This study has several limitations. The experiments were conducted mainly under controlled laboratory conditions, with specific instrument configurations and motion trajectories; performance under stronger occlusion, larger instrument sets, and more varied clinical conditions remains to be evaluated. Because of the available experimental conditions, the illumination-interference experiment introduced additional white-LED light only. No separate infrared interference source was tested. The observed detection stability therefore cannot establish robustness to external infrared illumination. Future work will evaluate the method under controlled infrared interference and more representative clinical imaging conditions, alongside end-to-end tracking latency and multi-instrument performance.
